# Decoding the role of DNA sequence on protein-DNA co-condensation

**DOI:** 10.1371/journal.pcbi.1013829

**Published:** 2025-12-18

**Authors:** Rohit Kumar Singh, Pinaki Swain, Mahipal Ganji, Sandeep Choubey

**Affiliations:** 1 The Institute of Mathematical Sciences, CIT Campus, Tharamani, Chennai, India; 2 Homi Bhabha National Institute, Training School Complex, Anushaktinagar, Mumbai, India; 3 Department of Biochemistry, Indian Institute of Science, Bangalore, India; Korea Institute for Advanced Study, KOREA, REPUBLIC OF

## Abstract

The compaction of DNA by phase-separating, DNA-binding proteins has emerged as a key mechanism for organizing chromatin and shaping genome architecture. Although experimental studies have provided insights into the governing principles of such protein-DNA co-condensation, how DNA sequence affects this process remains unclear. Guided by experimental observations, we develop a simple polymer-based model of protein-DNA co-condensation that explicitly accounts for sequence-dependent protein binding. Using coarse-grained Brownian dynamics simulations, we demonstrate that, in the case of a homogeneous DNA, only one condensate forms in equilibrium. In sharp contrast, DNA sequence heterogeneity can result in the coexistence of multiple condensates. Interestingly, we find that interfacial DNA binding affinity controls capillary forces generated by protein-DNA condensates, offering a potential mechanism to regulate chromatin structure and 3D genome organization. To demonstrate the usefulness of our modeling framework, we compare the simulation results against published data for the condensation of DNA via Dps, Sox2, and HP1. We find that DNA sequence dictates the condensation of Sox2 and HP1 with DNA. Overall, our framework provides mechanistic insights into how DNA sequence affects protein-DNA co-condensation and paves the way for developing a deeper understanding of genome organization.

## Author summary

Cells rely on a variety of proteins to compact and organize their DNA, enabling them to shape genome architecture and regulate gene expression. Many of these DNA-binding proteins can phase-separate with DNA, but how the underlying DNA sequence influences this behaviour remains elusive. Here, we use a minimal model to study protein-mediated DNA condensation along an end-tethered DNA molecule. Our Brownian dynamics simulations show that non-specific protein binding produces a single condensate at equilibrium, whereas sequence-dependent binding can generate multiple condensates of varying sizes, governed by the interplay between DNA–protein and protein–protein interaction strengths. We further find that the DNA–protein affinity at the condensate interface controls the capillary forces exerted on bare DNA. By comparing our results with experimental data for Dps, Sox2, and HP1, we show that DNA sequence strongly affects the condensation of Sox2 and HP1 but has minimal influence on Dps. Together, these findings provide mechanistic insight into how DNA sequence shapes protein–DNA condensation and advance our understanding of genome reorganization.

## 1 Introduction

The genome is spatially organized within the cell nucleus through three-dimensional (3D) compaction, a higher-order architecture essential for regulating key cellular processes such as gene expression, DNA replication, and repair [1–4]. This organization arises from hierarchical chromatin folding, which forms distinct yet pervasive features like loops, domains, and compartments across various length scales [4,5]. In prokaryotes, Nucleoid-Associated Proteins (NAPs) compact DNA to maintain nucleoid structure, thereby modulating DNA accessibility to transcription machinery and directly impacting global gene expression [6]. Despite considerable progress, the physical mechanisms by which DNA-binding proteins compact the genome and regulate gene expression in vivo remain less-understood. Unfurling the principles that govern genome architecture and its dynamic remodeling remains a central goal in regulatory biology.

Recent *in vitro* experiments suggest that transcription factors and DNA can undergo co-condensation through the collective behavior of thousands of proteins and DNA, driven by protein-protein and protein-DNA interactions [7–10]. The resulting protein-DNA co-condensate ensnares a specific amount of DNA, exerting force on the surrounding free DNA, known as capillary force [10–13]. Capillary forces, arising from the interfacial physics of protein-DNA condensates, have the potential to serve as a mechanism for regulating and reshaping chromatin. This capability holds promise in influencing the 3D architecture of the genome.

**Fig 1:**
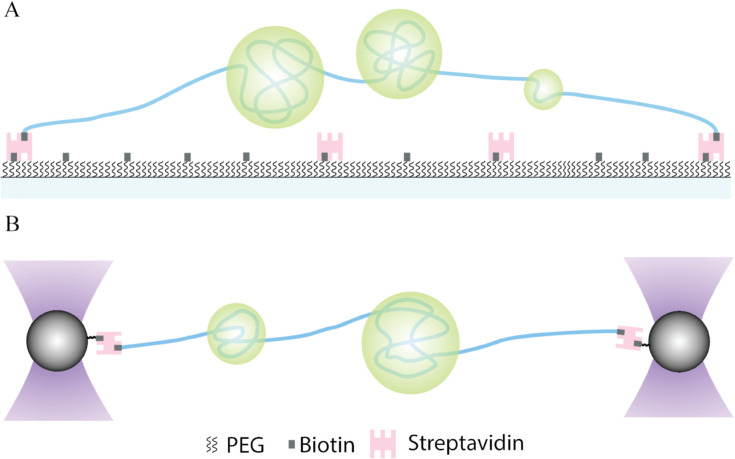
Experimental systems to study protein-DNA co-condensation. **A** Schematic of experimental setup wherein the ends of a single DNA molecule (blue) are tethered to the glass slide using biotin-streptavidin interactions. Proteins (green) phase-separate with DNA to form protein-DNA co-condensates. **B** Schematic of the optical tweezers assay: a DNA molecule (blue) is held between two optically trapped beads (black) via biotin–streptavidin interactions.

Experimental studies have investigated protein-DNA co-condensates and their impact on capillary forces by employing optical tweezers and coverslip-based assays, as shown in Fig 1. For instance, Quail et al. utilized total internal reflection fluorescence (TIRF) microscopy to explore the co-condensation of λ-phage DNA and the pioneer transcription factor protein FoxA1 [11]. Their findings revealed that the FoxA1-DNA co-condensate could generate forces on the order of 0.2 piconewton (pN). Another study highlighted that PARP1-DNA co-condensates generate capillary forces in the sub-piconewton range [12]. Intriguingly, a more recent investigation demonstrated that the pluripotent factor Sox2 can generate up to 7 pN of capillary forces [10].

These experiments provide insights into force–extension behavior as well. While such measurements for individual DNA molecules have been well-characterized [14,15], the presence of DNA-binding proteins has inspired extensive study over the past two decades. In untethered DNA, proteins can link distant segments, leading to collapse at high concentrations through a mechanism known as bridging-induced phase separation (BIPS) [16–20]. Notably, BIPS models typically neglect direct interactions between the proteins themselves. Introducing protein–protein interactions adds an additional layer of complexity by incorporating effects such as surface tension. However, most existing work has focused on protein–protein interactions either in untethered DNA systems [21–24] or in homogeneous tethered DNA setups [25], leaving aspects of these interactions underexplored.

An intriguing observation from several protein–DNA condensation studies is the formation of multiple discrete condensates along a single DNA molecule. For example, Sox2 was reported to form around 4–5 condensates [10], while HP1 typically formed 2 condensates along λ-phage DNA [26]. From a thermodynamic standpoint, phase separation of self-attracting molecules is expected to produce a single condensate at equilibrium [27,28], yet a clear theoretical framework explaining the persistence of multiple condensates remains incomplete. Similar behavior has also been documented in live cells, where multiple condensates coexist within the same system [29–35]. To address this, theoretical studies have approached the problem from both equilibrium perspectives [36–43] and non-equilibrium models incorporating chemical reactions coupled to phase separation [44–48]. Notably, Brackley et al. demonstrated that bridging-induced phase separation (BIPS) alone can give rise to multiple protein clusters along DNA, even with non-specific protein binding [17]. In this work, we investigate how the interplay between sequence-specific protein–DNA interactions and protein–protein interactions shapes the formation and spatial organization of multiple condensates along DNA, as observed in recent co-condensation experiments [10,26].

While a dialogue between theoretical and experimental studies have advanced our understanding of protein–mediated DNA compaction, the role of DNA sequence heterogeneity in shaping capillary forces and controlling the number and size of co-condensates remains poorly understood. The goal of this study is to develop a modeling framework to systematically investigate how DNA sequence variability influences protein–DNA co-condensation. To this end, we introduce a simple coarse-grained model that explicitly incorporates sequence-dependent protein binding, tailored to capture two common *in vitro* experimental setups: optical tweezer and coverslip-based assays. Using Brownian dynamics simulations, we show that a homogeneous DNA polymer yields a single condensate at equilibrium. In contrast, sequence heterogeneity promotes the coexistence of multiple condensates with variable sizes along the same DNA molecule. We demonstrate the utility of our framework by comparing simulation results with experimental observations of Dps, Sox2, and HP1 co-condensation with DNA. Our analysis reveals that while Dps binds DNA independently of sequence, Sox2 and HP1 co-condensates are highly sequence-sensitive. Furthermore, we find that the binding affinity of DNA at protein–DNA interfaces governs the magnitude of capillary forces, offering a potential mechanism to tune genome-scale mechanical forces through local DNA sequence changes. In summary, our framework enables the interpretation of in vitro co-condensation experiments and reveals how DNA sequence heterogeneity shapes protein–DNA condensation.

## 2 Model

To explore the impact of DNA sequence on the co-condensation of protein-DNA complexes and the subsequent generation of capillary forces, we propose a simple polymer-based model for protein-DNA binding. This model explicitly considers both protein-protein interactions and sequence-dependent protein-DNA interactions. The parameters of the model are chosen to mimic different experimental systems (see the Materials and Methods for details). In addition, the parameters used in our study are summarized in Table 1, with corresponding references (See Subsection 5.2 of the Materials and Methods section for more details).

**Table 1:** Model parameters.

Model parameters	Value used in this study	Consistent with prior studies
Protein concentration ($\rho_{p}$)	40–130 μM	Larson et al. [7], and Keenen et al. [26]
Protein–protein interaction strength ($\epsilon_{PP}$)	1.50–2.25 *k*_*B*_*T*	Sommer et al. [22], Swain et al. [24], Satheesh et al. [80], and Takaki et al. [98]
DNA–protein interaction strength ($\epsilon_{MP}$)	0.1–4.0 *k*_*B*_*T*	Morin et al. [8], Renger et al. [9], Brackley et al. [17], Ryu et al. [20], and Satheesh et al. [80]

**Fig 2:**
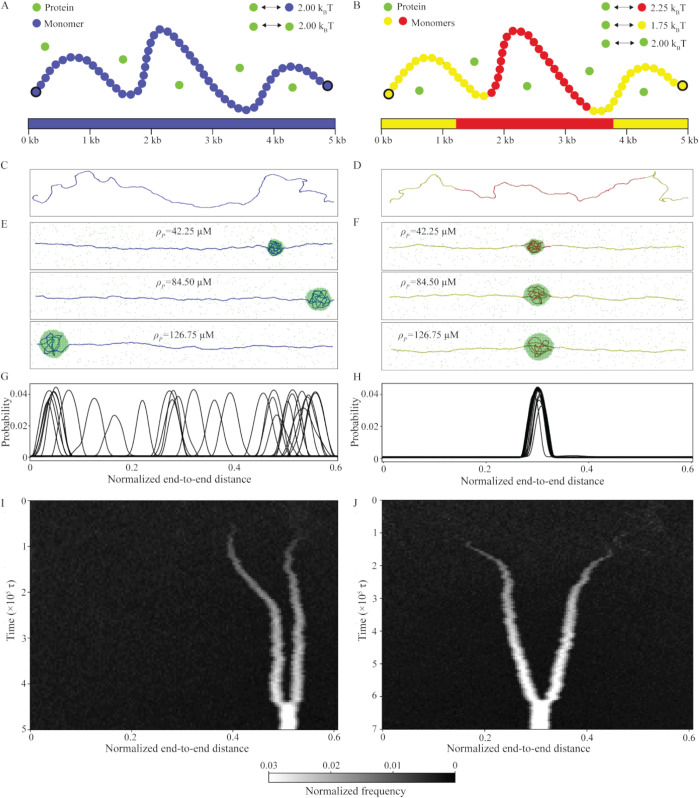
DNA sequence dictates the position of the condensates. **A** Schematic represents a homogeneous DNA model; each monomer (blue) binds to proteins (green) with an identical binding affinity (2 *k*_*B*_*T*). **B** Schematic represents heterogeneous DNA I model; monomers (red) at the center bind proteins with an affinity of 2.25 *k*_*B*_*T*, whereas monomers (yellow) at the periphery have an interaction strength of 1.75 *k*_*B*_*T*. Proteins bind to each other with an interaction strength of 2 *k*_*B*_*T*. **C**, **D** Snapshots of DNA configurations for homogeneous and heterogeneous DNA I, in the absence of proteins, are shown. **E**, **F** Snapshots of protein-DNA co-condensates in equilibrium for the two models are shown. To generate the plots, we fixed the normalized end-to-end distance ($R_{e}^{\prime }=0.6$) and varied $\rho_{p}$. **G**, **H** Probability distribution of DNA and proteins along the longitudinal axis at $R_{e}^{\prime }$ = 0.6, for different bulk protein concentrations ($\rho_{p}$), shown for homogeneous and heterogeneous DNA I, respectively. For each $\rho_{p}$ value, data from five independent replicates are included in the plots. **I**, **J** Representative kymographs for position of condensates along the contour as a function of time for homogeneous DNA ($R_{e}^{\prime }$=0.6, $\rho_{p}$= 109.9 μM) and heterogeneous DNA I ($R_{e}^{\prime }$=0.6, $\rho_{p}$= 93.01 μM) are shown.

Informed by *in vitro* studies, as shown in Fig 1, we consider a 5 kb-long DNA as a semiflexible polymer consisting of *N*_*m*_ = 500 monomers, tethered at both ends within a cuboidal box. In accordance with previous studies [49–52], the persistence length of DNA is considered to be 150 bps, which translates to 15 monomers in our model. The DNA is in contact with proteins. We assume that each monomer corresponds to 10 base pairs which is the approximate footprint of a family of transcription factors in bacteria [53–55]. Our study focuses on transcription factors that bind to shorter sequences. For example, Sox2 binds to 7 bp DNA sequences [56], while Klf4 binds to 7 to 10 bp [57]. We assume that the proteins are spherical beads with dimensions identical to the monomers. The sequence heterogeneity of DNA is represented by distinct arrangements of monomers along the DNA contour, where each monomer exhibits varying binding affinity for the proteins. To dissect the impact of DNA sequence on protein-DNA co-condensation, first, we introduce a null model of homogeneous DNA. In this model, each monomer has an identical binding affinity of 2 *k*_*B*_*T* for proteins (see Fig 2A) [8]. To investigate how DNA sequence heterogeneity influences protein–DNA co-condensation, we analyze three DNA models of increasing complexity. In the first two heterogeneous models, DNA is represented as a polymer composed of two distinct types of monomers, each corresponding to a 10 bp double-stranded DNA segment that characterizes a specific protein-binding motif. These monomer types reflect strong and weak binding affinities for proteins, capturing a binary classification of nucleotide segments that represents biologically relevant binding landscapes. For example, nucleoid-associated proteins like Lsr2 [58] and H-NS [59] exhibit strong binding to AT-rich regions and weaker affinity for GC-rich sequences. 1) Heterogeneous DNA I, in this model the DNA is represented as an ABA block copolymer based on differential attractive interaction with proteins. Here DNA consists of a high-affinity region spanning 250 monomers at the center, with a protein-DNA binding affinity of 2.25 *k*_*B*_*T*. This high-affinity region is surrounded by low-affinity regions on either side, each containing 125 monomers, with a protein-DNA binding affinity of 1.75 *k*_*B*_*T*. This ensures that the average protein-DNA binding affinity remains identical to the homogeneous case (see Fig 2B). 2) Heterogeneous DNA II model consisting of two blocks of high-affinity monomers separated by low-affinity monomers, leads to multiple condensates at equilibrium when protein-DNA interactions dominate over protein-protein interactions (see Fig 4 and SI). 3) Partial λ-DNA, to model a biologically realistic DNA, we consider the last 5 kb of the lambda DNA (see Fig 5A and Methods section). For simplicity, we assume that each protein has a preference for binding to AT-rich regions. Indeed, there exist numerous proteins, in bacteria and higher organisms, that broadly bind AT or GC-rich sequences [58,60,61]. We assign a corresponding interaction energy to each monomer based on the AT content of each 10-base sequence (see the Materials and Methods and Supplementary Information (SI), including Table A-D in S1 Text, for details).

Following the experiments, we tune the normalized end-to-end distance of DNA and the bulk protein concentration. Normalized end-to-end distance ($R_{e}^{\prime }$) is defined as the ratio of the distance between the tethered ends (*R*_*e*_) and the contour length of the DNA (*s*). Bulk protein concentration ($\rho_{p}$) is the initial concentration of proteins introduced in the simulation box in units of μM. We employ coarse-grained Brownian dynamics simulation to study sequence-dependent protein-DNA condensation (see the Methods and SI for details).

## 3 Results

### 3.1 DNA sequence governs the capillary forces emanating from protein-DNA co-condensates

We seek to uncover the effect of DNA-sequence heterogeneity on the formation of protein-DNA co-condensates and the resultant capillary forces. In particular, we consider two models: homogeneous DNA (Fig 2A) and heterogeneous DNA I (Fig 2B). For a detailed discussion on the choice of parameters, see the methods section and Sec. S1. Our DNA model is homogeneous or heterogeneous only with respect to protein-binding. In the absence of proteins, both the sequences remain extended as shown in Fig 2C and 2D. In contrast, proteins form droplets in the absence of DNA around $\rho_{p}$=190 μM (Fig F in S1 Text). When proteins are introduced along with the DNA, we observe phase separation at $\rho_{p}$ = 42.25 μM for both free-end DNA and double-tethered DNA (Fig F, panels B and C in S1 Text). This observation is consistent with previous theoretical [22] and experimental studies [26]. For subsequent investigation, we keep the normalized end-to-end distance ($R_{e}^{\prime }$) fixed and systematically increase the bulk protein concentration ($\rho_{p}$). For both homogeneous DNA and heterogeneous DNA I, we observe an increase in condensate size with increasing bulk protein concentration ($\rho_{p}$), but the condensate remains spherical (Fig 2E and 2F). However, we see a change in the shape of the condensate with variation in protein-protein interaction at constant $\rho_{p}$. When protein-protein interaction is lower than the protein-DNA binding affinity, elongated condensates emerge (Fig G, panels A and B in S1 Text). As we increase protein-protein interactions, the shape of condensates becomes more and more spherical (Fig G, panels C and D in S1 Text).

For a homogeneous DNA, a single condensate tends to form near the tethered ends as shown in Fig 2G (Fig H, panels A and B in S1 Text). To verify this positional bias in homogeneous DNA, we perform a longer simulation of a flexible homopolymer of shorter length. We find that a single condensate is more likely to remain close to the center in equilibrium due to larger conformational fluctuations of the polymer in the center (Fig H, panels C and D in S1 Text). Thus, the end-position preference observed in Fig 2G can be attributed to computationally expensive simulation time to sample different configurations for a long chain. In the case of the heterogeneous DNA I, a single condensate forms in equilibrium at the high-affinity region of the DNA, therefore maximizing their interactions with proteins, as illustrated in Fig 2H. While multiple co-condensates nucleate along the DNA for homogeneous DNA (Fig 2I) and heterogeneous DNA I (Fig 2J) at higher protein concentrations, these condensates fuse and coarsen over time to become a single condensate in equilibrium. The Laplace pressure facilitates this coarsening process, as a single condensate minimizes the surface area compared to multiple smaller condensates. Note that Laplace pressure is the pressure difference across the interface between the dense and dilute phases of condensates. It arises due to surface tension and is inversely proportional to the radius of a condensate [62].

**Fig 3:**
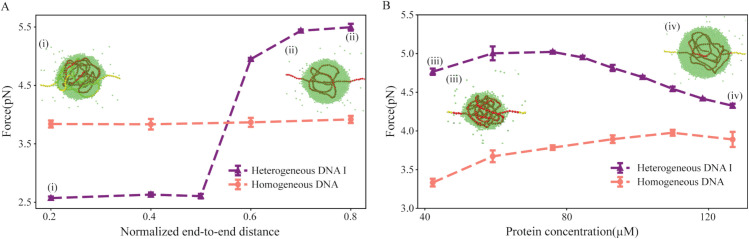
Average interfacial DNA affinity dictates capillary forces. **A** and **B** Capillary force is plotted as a function of $R_{e}^{\prime }$ at $\rho_{p}$=84.50 μM and as a function of $\rho_{p}$ at $R_{e}^{\prime }$=0.6 for homogeneous (salmon) and heterogeneous DNA I (purple), respectively. Each point in the plots represents the average of five independent replicates, with error bars denoting the standard deviation. The snapshots show condensates for heterogeneous DNA I where red represents high-affinity monomers while yellow represents low-affinity monomers. Proteins are shown in green. Snapshots (left to right) show condensates for the following parameter sets: (i) $R_{e}^{\prime }$= 0.2 and $\rho_{p}$= 84.50 μM, (ii) $R_{e}^{\prime }$= 0.8 and $\rho_{p}$= 84.50 μM, (iii) $R_{e}^{\prime }$= 0.6 and $\rho_{p}$= 42.25 μM and (iv) $R_{e}^{\prime }$= 0.6 and $\rho_{p}$= 126.75 μM.

Next, we investigate the effect of protein-DNA co-condensation on capillary force as a function of bulk protein concentration ($\rho_{p}$) and normalized end-to-end distance of DNA ($R_{e}^{\prime }$). See the Materials and Methods, subsection 5.5, for details on the computation of capillary force. For homogeneous DNA, we observe a marginal increase in capillary forces on bare DNA as we increase $\rho_{p}$ and $R_{e}^{\prime }$ (Fig 3A and 3B). To validate that the force on bare DNA indeed arises at the interface of the condensate, we calculate the potential energy for each monomer along the contour for homogeneous DNA at $R_{e}^{\prime }$= 0.2 and $\rho_{p}$ = 84.50 μM (Fig D in S1 Text). Indeed, the potential energy sharply changes for the monomers present at the interface, confirming that the force on the bare DNA is generated at the interface of the protein-DNA co-condensate. For heterogeneous DNA I, the capillary force exhibits a non-linear behavior as we tune $R_{e}^{\prime }$. Initially, an increase in $R_{e}^{\prime }$ leads to a small increase in capillary force, followed by a sharp jump at $R_{e}^{\prime }∼0.5$ (Fig 3A). On the other hand, as we increase $\rho_{p}$, an opposite trend is observed (Fig 3B). The capillary force initially remains constant as we increase $\rho_{p}$, followed by a gradual decrease after $\rho_{p}$ = 59.15 μM. The behavior of capillary force for heterogeneous DNA I is dictated by the affinity of the interfacial DNA. When $R_{e}^{\prime }$ exceeds a certain threshold (here 0.5), the higher affinity DNA (shown in red) gets exposed at the interface of the condensate (between compacted DNA and bare DNA), resulting in an increased force exerted on bare DNA (Fig I, panel A in S1 Text). In contrast, with an increase in $\rho_{p}$, condensate volume increases linearly (Fig I, panel B in S1 Text), which leads the condensate interface to be exposed to low-affinity DNA (shown in yellow). As a result, there is a decrease in the capillary force exerted on the bare DNA (Fig 3B and Fig I, panel C in S1 Text). Overall, our results demonstrate that the protein binding affinity of the interfacial DNA dictates the capillary forces. In the ensuing sections, we probe how different DNA sequences affect the capillary forces.

### 3.2 DNA sequence heterogeneity leads to the formation of multiple condensates

**Fig 4:**
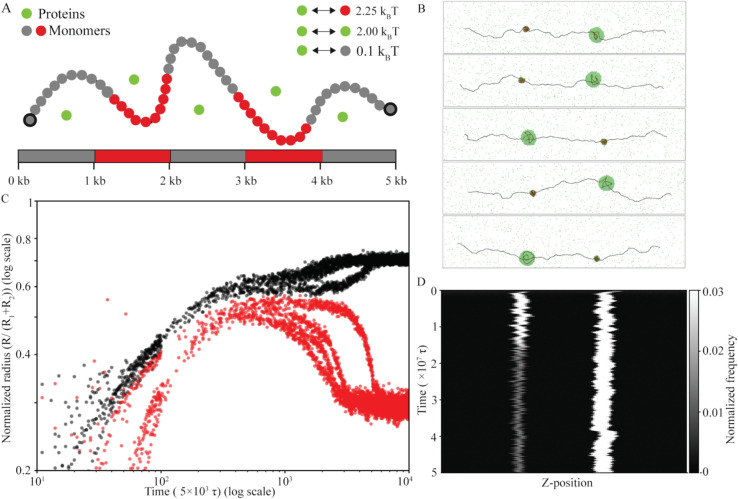
DNA sequence heterogeneity leads to the co-existence of multiple protein-DNA condensates at equilibrium. **A** Schematic represents heterogeneous DNA II model: two blocks of high-affinity monomers (2.25 *k*_*B*_*T*, 100 monomers in red) are separated by a block of low-affinity monomers (0.1 *k*_*B*_*T*, grey) of identical length. Proteins (green) interact with each other with an interaction strength of 2 *k*_*B*_*T*. **B** Snapshots shown for cases with two condensates co-existing at equilibrium for all five independent realizations at $R_{e}^{\prime }=0.6$ and $\rho_{p}$=84.50 μM. High-affinity monomers, low-affinity monomers, and proteins are shown in red, grey, and green respectively. **C** Scaled radius of the larger droplet (black) and smaller droplet (red) is shown for all the five trajectories quantifying the arrested coarsening. **D** Representative kymograph showing visualization of coarsening kinetics for one initial condition.

To further investigate the impact of DNA sequence heterogeneity on protein–DNA co-condensation, particularly how DNA-protein and protein-protein interaction strengths affect the number and size of co-condensates, we examine the heterogeneous DNA II model (Fig 4A). This model features two blocks of high-affinity monomers separated by a block of low-affinity monomers (see the Model section and SI for details). Interestingly, the relative strengths of DNA-protein and protein-protein interactions lead to distinct regimes with specific droplet size and number. We consider three parameter regimes: (1) when protein–protein interactions are much weaker than protein–DNA interactions, (2) when they are comparable but slightly weaker, and (3) when protein–protein interactions are much stronger than protein–DNA interactions. In all cases, the protein concentration ($\rho_{p}$) is fixed at 84.50 μM and the normalized end-to-end distance ($R_{e}^{\prime }$) is maintained at 0.6. When protein-protein interactions are much weaker than DNA-protein interactions (Regime 1), two equal-sized DNA–protein co-condensates form in equilibrium, with their size determined by the DNA bound in each (Fig J, panels A, C, and E in S1 Text). When the two interaction strengths are comparable (Regime 2), two condensates form initially, but Ostwald ripening drives one to grow at the expense of the other, yielding unequal sizes at equilibrium (Fig 4B and 4C). However, the stronger protein-DNA interactions keep the two condensates stable. When protein-protein interactions exceed DNA-protein interactions (Regime 3), proteins prefer to cluster with each other more than binding DNA monomers, leading the system to coarsen into a single large co-condensate with one of the high-affinity regions along the DNA, thereby minimizing the total surface energy (Fig J, panels B, D, and F in S1 Text). Below, we explore the second parameter regime in greater detail.

To ascertain that the two-condensate state is an equilibrium state, we look at the coarsening kinetics in Fig 4C. We follow the time evolution of the normalized radii of the two condensates. Fig 4C shows that two condensates are roughly of the same size till $∼10^{6}\tau$, after which one of the condensates (black) grows at the expense of the other (red). Notably, both the droplets show saturation in size at a late time ($∼10^{7}\tau$), indicating that coarsening is arrested. Note that we see arrested coarsening irrespective of the initial condition, which suggests that the two-condensate state is an equilibrium state. The representative kymograph for one of the realizations confirms that coarsening is arrested after $∼10^{7}\tau$ (Fig 4D).

**Fig 5:**
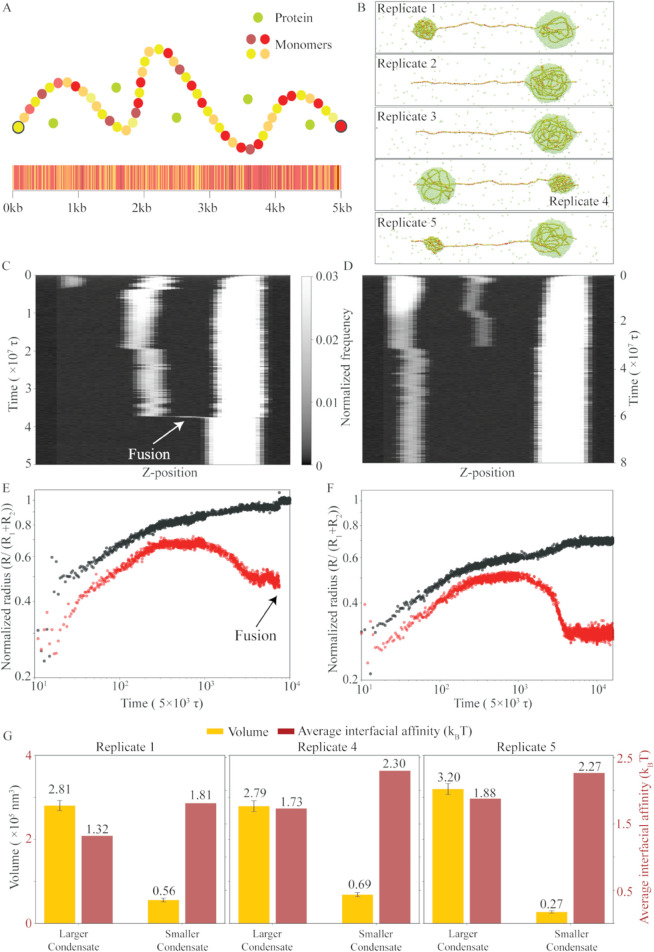
Sequence heterogeneity in partial λ-DNA engenders multiple condensates as metastable states. **A** Schematic represents the Partial λ-DNA: Eleven kinds of monomers (yellow to maroon) are introduced, based on AT content of 10 base pairs, modeled as one monomer. The monomer-protein binding affinities vary in the range of 0.1 to 4 *k*_*B*_*T* depending on the AT content of the corresponding 10 bp motif that represents the monomer (see the model section). Proteins (green) bind to each other with an affinity of 2 *k*_*B*_*T*. **B** Snapshots shown for five independent realizations at $R_{e}^{\prime }=0.2$ and $\rho_{p}$=84.50 μM (top to down). **C, D** Representative kymograph showing visualization of coarsening kinetics for replicate 2 and 5. **E** Scaled radius of the larger condensate (black) and smaller condensate (red) is shown for replicate 2 which leads to a single condensate at equilibrium. **F** Scaled radius of the larger condensate (black) and smaller condensate (red) is shown for replicate 5 which shows coexistence of two condensates as a kinetically trapped state. **G** Bar plots show the average interfacial affinity (maroon) and volume (orange) for two condensates observed in the case of replicates 1, 4, and 5.

Next, we study a biologically realistic DNA sequence, the Partial λ-DNA (Fig 5A), to gain a deeper understanding of how DNA sequence impacts the number of protein-DNA co-condensates. For a detailed discussion of this model, see the model section and Table D in Sec. S1 of the S1 Text. Fig 5B shows that the number of condensates varies for a fixed $R_{e}^{\prime }$ and $\rho_{p}$ in different realizations. While replicates 2 and 3 in Fig 5B show a single condensate as in the homogeneous DNA model, replicates 1, 4, and 5 show two condensates as in the heterogeneous DNA II model. This suggests that a more heterogeneous DNA sequence like the Partial λ-DNA sequence may show initial condition dependency, and get trapped in a long-lived metastable state of multiple droplets even though the equilibrium state is that of a single droplet.

We confirm the initial condition dependency by studying the kinetics leading to a single and double condensate state for two representative replicates. The kymograph in Fig 5C gives a visualization of the early-time coarsening followed by the fusion event for replicate 2, which leads to a single condensate. In contrast, we do not see such a fusion event for the double-condensate case observed in replicate 5 (Fig 5D). To quantify the coarsening kinetics, we look at the time evolution of the size of the condensates. For the single condensate state observed in replicate 2, the normalized radii of both the condensates grow till $∼10^{6}\tau$, after which condensate 1 (shown in black) grows while condensate 2 (shown in red) shrinks. However, at ∼4 × $10^{7}\tau$, condensate 2 fuses with condensate 1, leading to one condensate at equilibrium (Fig 5E). The normalized radii of the two condensates, formed after early-time growth and coarsening, saturate at late times, as shown in Fig 5F. It is possible that for an infinitely long simulation, replicate 5 shows a single condensate. Such a long-lived state of local equilibrium is known as a metastable state and can manifest in experiments due to time scale limitations.

To further investigate the origin of metastable states, we analyze the volumes and average interfacial affinities of individual condensates in replicates that exhibit two condensates (Fig 5G). In all such cases, we find that the smaller condensate consistently displays a higher average interfacial affinity than the larger one. In the next section, we show that interfacial affinity plays a key role in modulating the capillary forces in the Partial λ-DNA sequence across all *R*_*e*_ and $\rho_{p}$ values examined in our study.

### 3.3 Alterations in the local affinity of interfacial DNA regulate the global capillary forces

**Fig 6:**
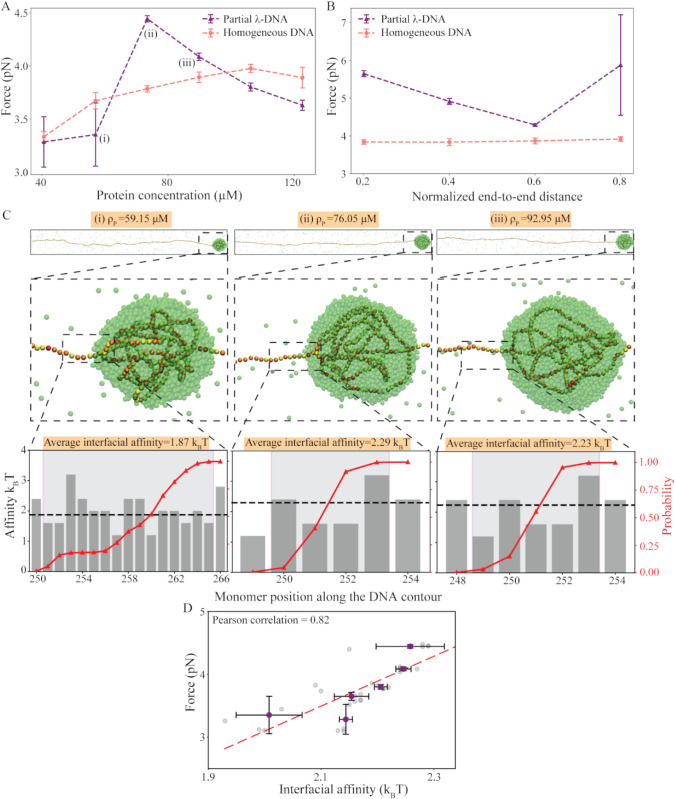
Local interfacial DNA affinity dictates global capillary forces. **A** Capillary force as a function of $\rho_{p}$ at fixed $R_{e}^{\prime }$=0.6. **B** Capillary force as a function of $R_{e}^{\prime }$ at fixed $\rho_{p}$. **C** (top panel) Snapshots of condensates at concentrations marked as (i), (ii), and (iii) in **A**. Monomers are shown in yellow to red in ascending order protein binding affinity and the proteins are shown in green. **C**(bottom panel) Bar plots represent monomer-protein binding affinities. The grey-shaded region shows the interface. The red curve shows the probability of the monomer being inside the condensate. **D** capillary force correlates with average interfacial affinities. Grey points show the capillary forces and interfacial affinities for 30 individual replicates at the six $\rho_{p}$ values in **A**. The purple points show the mean and standard deviation at each $\rho_{p}$. The red dashed line is a fit to the mean data.

To further elucidate the role of DNA sequence in shaping the capillary forces exerted by protein–DNA co-condensates, we measure the capillary force for the partial λ-DNA model as a function of protein concentration ($\rho_{p}$) at a fixed extension of $R_{e}^{\prime }=0.6$, and as a function of extension ($R_{e}^{\prime }$) at a fixed protein concentration $\rho_{p}=84.50$ μM. For both cases, the capillary force exhibits an intriguing non-monotonic behavior (see Fig 6A and 6B). While the null homogeneous DNA model shows a small and monotonic increase in force with $\rho_{p}$, the partial λ-DNA model shows an initial increase followed by a decrease in force.

To understand the non-monotonic behavior of capillary force, we analyze the average interfacial affinities of the DNA sequences at the protein concentrations showing non-monotonic variation. We observe a  2 pN change in capillary force with an increase in $\rho_{p}$ from 59.15 μM to 76.05 μM (marked as (i) and (ii) in Fig 6A). Although the DNA segments inside the condensate remain nearly identical (snapshots in Fig 6C), the sequences at the interface get altered, which leads to significant differences in the capillary forces (bottom panels in Fig 6C). A comparison of the interfaces shows that the inclusion of a high-affinity motif at the interface for $\rho_{p}$= 76.05 μM, leads to pinning of the condensate along the DNA contour, resulting in a smaller interface width (Fig 6C bottom panel). This results in an increase in the average interfacial affinity from 1.87 *k*_*B*_*T* to 2.29 *k*_*B*_*T*. Further increase in $\rho_{p}$ to 92.95 μM (marked as (iii) in Fig 6A), results in a decrease in force and a corresponding decrease in average interfacial affinity to 2.23 *k*_*B*_*T*.

To establish the role of the interface in dictating the capillary forces, we plot the average interfacial affinity for all the 30 simulations conducted for the partial λ-DNA sequence at six $\rho_{p}$ values (Fig 6D). Next, we calculate the mean and standard deviation of the average interfacial affinity over the five replicates at each $\rho_{p}$. Indeed, we observe a high correlation of 0.82 between the capillary forces and average interfacial affinity over the five simulations. We observe a similar correspondence between the interfacial affinity and capillary force with variation in $R_{e}^{\prime }$ at fixed $\rho_{p}$ (Fig K in S1 Text).

Our findings indicate that small variations in motif-level binding affinity, arising from mutations or methylation patterns, can substantially alter the global capillary forces. In fact, if the affinity of protein binding to a given motif at the interface of a co-condensate changes by even one *k*_*B*_*T*, two-fold changes in global forces are observed in simulations (Fig 6).

### 3.4 HP1 and Sox2 exhibit DNA sequence-dependent co-condensation

The results obtained from our simulations can be harnessed to interpret recent experimental results [10,11,26] on protein-DNA co-condensation. In particular, our results show that a homogeneous DNA sequence, where a protein binds with uniform affinity along the entire length of DNA, leads to only a single condensate at equilibrium across all tested parameter regimes. In contrast, sequence heterogeneity in DNA can give rise to multiple condensates along the DNA, with their size and number determined by the interplay between protein–protein and protein–DNA interactions. A key implication of our work is that the emergence of multiple, spatially separated condensates requires underlying sequence heterogeneity. Thus, if experiments reveal multiple condensates at long times, the homogeneous DNA model can be ruled out, indicating that sequence features dictate protein–DNA co-condensation. In this light, we analyze the number of co-condensates along the DNA for a set of proteins: the nucleoid-associated protein Dps, which condenses DNA through bridging interactions [63]; the nucleosome-binding pioneer transcription factor Sox2 [10]; and HP1, a key driver of heterochromatin formation [26].

**Fig 7:**
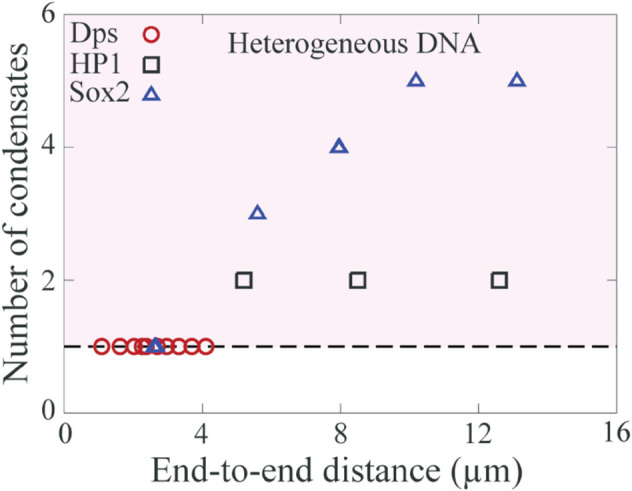
A comparison between simulation and experimental results. Scatter plot shows the number of condensates as a function of end-to-end distance for Dps (taken from Figure 4 of [63]), Sox2 (taken from Figure 1b and 3i of [10]), and HP1 (taken from Figure 4B of [26]). The dashed line, depicting the condensate number of unity, reflects sequence-independent co-condensation, whereas the region shaded in pink shows sequence-dependent co-condensation of protein and DNA.

We first examine the co-condensation of Dps, as observed in a recent study [63], which reported the formation of a single condensate across varying normalized end-to-end DNA distances (Fig 7). This behavior is consistent with predictions from both the homogeneous and Partial λ-DNA models, suggesting that Dps binds DNA in a sequence-independent or only weakly sequence-dependent manner, with co-condensation primarily driven by protein-protein interactions. This interpretation aligns with earlier findings that Dps exhibits sequence-independent DNA binding [63,64]. In contrast, Sox2–DNA co-condensation shows markedly different behavior. Recent experiments reveal the formation of multiple distinct co-condensates along the DNA (Fig 7), which contradicts the predictions of the homogeneous model (Fig 2), thereby falsifying it. Instead, these experimental observations are well captured by our heterogeneous DNA models, highlighting the potential role of DNA sequence in guiding Sox2-mediated co-condensation.

Next, we turn our attention to the co-condensation of HP1 with DNA. Hitherto the binding of HP1 to DNA has been considered as relatively sequence-independent [65]. Based on these previous studies, we hypothesize that the homogeneous DNA model would effectively capture the condensation of HP1 with DNA. Contrary to our expectations, HP1 forms two condensates (see Fig 7), with one located near a tethered end and another in the middle of the DNA, thereby falsifying our hypothesis [26]. A heterogeneous DNA model with sequence heterogeneity can explain the coexistence of multiple co-condensates at different locations along the DNA. Our analysis suggests that HP1 exhibits differential binding affinity to DNA.

Note that our models don’t distinguish between Sox2-Sox2, HP1-HP1 or Dps-Dps interactions. However, our work helps us distinguish between the three proteins in terms of their DNA-binding landscape. In other words, based on the different models of DNA we employ, the protein–protein interaction strength is the same for all three proteins, fixed at $\epsilon_{PP}=2$ *k*_*B*_*T*.; However, for Sox2 and HP1, the protein–DNA interaction strength varies along the DNA (e.g., $\epsilon_{AP}=0.1$, $\epsilon_{BP}=2.25$ *k*_*B*_*T*), in line with our heterogeneous DNA model II. For Dps, the interaction is uniform, $\epsilon_{AP}=2$ *k*_*B*_*T*, consistent with our homogeneous DNA model I. Thus, we do not explicitly consider the molecular identities of these proteins, but rather contrast homogeneous and heterogeneous protein–DNA binding landscapes, using Dps, HP1, and Sox2 as illustrative examples.

## 4 Discussion

Recent evidence suggests that phase separation of chromatin and DNA might play a crucial role in organizing the genome [7,66–77]. However, probing the biophysical principles of such phase-separated bodies *in vivo* has proved to be challenging. Alternatively, *in vitro* biochemical studies with purified transcription factors (TF) and DNA have shed light on protein-mediated condensation of DNA [9–11]. A series of such *in vitro* studies have shown that protein-protein and protein-DNA interactions lead to condensation of DNA [63,78,79]. However, the role of DNA sequence has thus far remained unclear. We present a simulation framework to investigate how DNA sequence influences protein–DNA co-condensation in double-tethered DNA systems.

To investigate how sequence heterogeneity influences capillary forces and the number of condensates, we use a model in which DNA is represented as a semiflexible polymer and proteins are modeled as interacting particles. We consider four different DNA models: (i) a homogeneous DNA model, where proteins bind uniformly without sequence preference; (ii) heterogeneous model I, having a central high-affinity binding region; (iii) heterogeneous model II, with two high-affinity regions separated by a low-affinity segment; and (iv) a partial λ-DNA model, representing a biologically realistic, heterogeneous binding landscape from a segment of λ-phage DNA. Note that the choice of parameters in these models is informed by earlier work on DNA-protein condensation (See Subsection 5.2 of the Materials and Methods section for more details). Using these models, we address two key questions: (1) How do capillary forces vary with end-to-end distance ($R_{e}^{\prime }$) and protein concentration ($\rho_{p}$) across different DNA sequences? and (2) What are the minimal sequence and energetic conditions necessary to support the formation of multiple distinct condensates?

**Fig 8:**
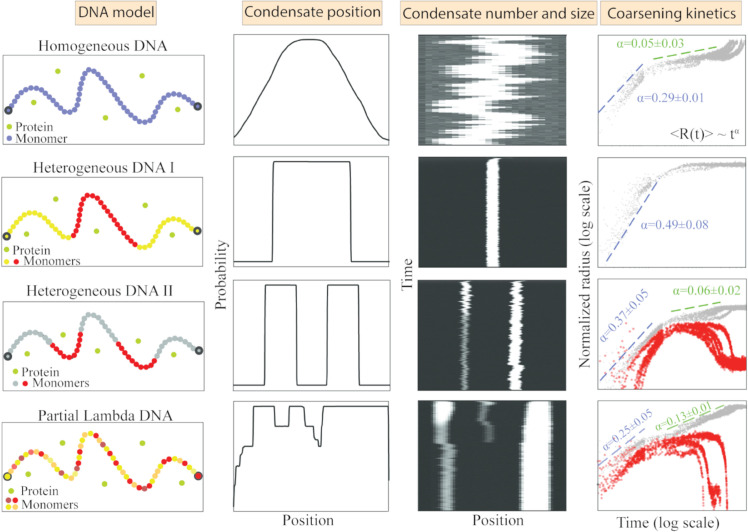
Key insights from simulations of DNA sequence-dependent protein–DNA Co-condensation. **First column** Cartoon representations of the four DNA models considered in our study. **Second column** Probability of each monomer being inside the condensate is plotted as a function of monomer position along the contour for a shorter homogeneous DNA (*N*_*m*_ = 50, $R_{e}^{\prime }$ = 0.8, and $\rho_{p}$= 84.5 μM), heterogeneous DNA I ($R_{e}^{\prime }$ = 0.6 and $\rho_{p}$= 84.5 μM), heterogeneous DNA II ($R_{e}^{\prime }$ = 0.6 and $\rho_{p}$= 84.5 μM), and partial λ-DNA ($R_{e}^{\prime }$ = 0.2 and $\rho_{p}$= 84.5 μM). **Third column** Representative kymographs are shown for all the models for the same conditions as in the second column. **Fourth column** Coarsening kinetics of a single final condensate is shown for five independent realizations of homogeneous DNA (*N*_*m*_ = 500, $R_{e}^{\prime }$ = 0.6, and $\rho_{p}$= 126.75 μM) and heterogeneous DNA I ($R_{e}^{\prime }$ = 0.6 and $\rho_{p}$= 126.75 μM). Coarsening kinetics of two droplets (larger condensate radius in grey and smaller condensate radius in red) are shown for heterogeneous DNA II ($R_{e}^{\prime }$ = 0.6 and $\rho_{p}$= 84.5 μM), and partial λ-DNA ($R_{e}^{\prime }$ = 0.6 and $\rho_{p}$= 126.75 μM). Panels are arranged as homogeneous DNA, heterogeneous DNA I, heterogeneous DNA II, and Partial λ-DNA from top to bottom.

We find that for a homogeneous DNA, even if multiple condensates form initially, they tend to coarsen over time, eventually resulting in a single condensate—a phenomenon known as Ostwald ripening. For the homogeneous DNA, the condensate position can be anywhere but with a maximum probability of being at the center (Fig 2). However, for the heterogeneous DNA I model, the presence of strong binding sites at the center pins the condensate position at that location. Although both models lead to a single condensate at equilibrium, the capillary forces resulting from co-condensation differ qualitatively between them. With variations in $R_{e}^{\prime }$, the homogeneous DNA model shows no change in the capillary force. In contrast, the heterogeneous DNA I model exhibits a switch-like behavior around $R_{e}^{\prime }∼0.5$ (Fig 3). This behavior can be explained by the composition of monomers at the condensate interface in the heterogeneous DNA I model. In particular, at $R_{e}^{\prime }∼0.5$, a transition occurs: below this point, low-affinity monomers occupy the interface, while above this threshold, high-affinity monomers take their place (Fig 3). Interestingly, interfacial affinity also accounts for variations in capillary force in biologically relevant sequences, such as Partial λ-DNA. For instance, capillary force exhibits a non-monotonic response as a function of protein concentration ($\rho_{p}$) (Fig 6A). Indeed, further analysis reveals a direct correlation between average interfacial affinity and capillary forces (Fig 6D). While the total interfacial energy reflects contributions from various DNA–protein, and protein-protein interactions, our findings suggest that even small changes in the identity of DNA monomers at the condensate interface—and the corresponding change in interfacial affinity (*I*_*a*_)—can effectively capture the observed variations in capillary forces. For simplicity, we restrict our measurement of capillary forces to simulations that result in a single condensate at equilibrium. However, the Partial λ-DNA sequence also shows initial condition dependency, i.e., at the same parameter values, we observe one or two condensates for different initial conditions. The two condensates observed in some replicates do not coarsen over long simulation time leading to long-lived metastable states (Fig 5). Further analysis shows that in the case of replicates showing two condensates, the smaller condensates contain high-affinity monomers at the interface (Fig 5G). In contrast, the heterogeneous DNA II model shows two condensates for all replicates, when DNA-protein interaction at the high-affinity sites dominates over the protein-protein interaction (Fig 4). We confirm these findings with coarsening kinetics analysis and see that the droplet size has saturated for all replicates. Our results indicate that interfacial affinity explains the minimal condition to sustain multiple condensates, as well as, the non-monotonicity observed in capillary forces for heterogeneous sequences. We summarize our results in Fig 8.

The distinction between homogeneous and heterogeneous DNA sequences is gradual rather than discrete. Heterogeneity arises from variations in sequence patterning and differences in protein-binding affinity among monomers, though protein-protein interactions also influence the biophysical properties of protein-DNA co-condensates, including their number and spatial positioning. Introducing correlated heterogeneity, such as alternating high- and low-affinity blocks, can localize condensate positions along the DNA even with small affinity differences, while uncorrelated variations make the average local affinity the main determinant of condensate position, as we observed for a nucleoid-associated protein Lsr2 [80].

Upon revisiting protein-DNA co-condensation data from previous experimental studies [10,11,26,63], we find that bacterial nucleoid-associated protein Dps compacts DNA in a sequence-independent or weakly sequence-dependent manner. In contrast, pioneer factor Sox2 and heterochromatin protein HP1 exhibit sequence-dependent binding to DNA [10,26,81]. Our findings for Dps are consistent with previous experimental studies [63,64]. Similarly, Sox2 is known to exhibit high sequence specificity; Sox2 recognizes 7 base pair (bp) of (e.g. CTTTGTT) DNA sequences [56,81,82]. Therefore, our discovery of the co-condensation of Sox2 being influenced by DNA sequence is not surprising. Remarkably, our re-analysis of a recent paper on HP1-mediated DNA condensation revealed the sequence-dependence of HP1 binding to DNA [26]. In particular, the existence of multiple condensates implies that DNA sequence governs HP1 condensation with DNA. This observation challenges the prevailing understanding of HP1 binding to DNA, which is generally considered to be relatively sequence-independent. Moreover, in the study involving Sox2-DNA co-condensation [10], the authors confirmed that the system reached equilibrium by monitoring the condensate intensities over an extended period until they plateaued. In contrast, the HP1-DNA co-condensation study did not include such equilibrium measurements; nevertheless, experiments using DNA with varying end-to-end distances consistently showed two condensates along the DNA [26]. For the HP1-DNA system, however, we cannot exclude the possibility that the system becomes trapped in a metastable state. Undoubtedly, further experiments are necessary to test the sequence dependence of HP1 binding to DNA and its contribution to DNA condensation, preferably involving controlled design of DNA sequences.

We have modelled proteins as spherical beads of the same size as DNA monomers, as detailed in the model section and supplementary information. While acknowledging that actual proteins have diverse structural features influencing their phase separation behavior, our coarse-grained approach captures essential features of sequence-dependent protein-DNA co-condensation. We find that the relative strengths of protein-protein and protein-DNA interactions govern key sequence-dependent effects. Our simplified model can capture experimental observations of proteins like Dps, HP1, and Sox2, reproducing known behaviors such as sequence-independent binding of Dps and sequence-dependent binding of Sox2. A recent study on the bacterial nucleoid-associated protein Lsr2 demonstrated that both the single-bead protein model, like the one used in our work, and a more detailed model incorporating Lsr2’s dimeric and multivalent features yield comparable results for the number and positions of Lsr2-DNA condensates, closely recapitulating experimental observations [80]. These results highlight that our simplified model can provide key mechanistic insights into experimental observation of protein-DNA co-condensation and shed light on genome compaction and gene regulation.

Biomolecular condensates in cells have traditionally been thought to form via bulk phase separation [83–87]. However, recent experiments suggest that often the formation of these condensates involves various surfaces inside cells such as DNA, microtubule, cytoskeleton, membranes, etc [88–90]. The interfaces associated with these surface-mediated condensates give rise to interfacial capillary forces [9,11,88]. However, capillary forces and their physiological implications remain relatively understudied. Our findings suggest that capillary forces arising from the interfacial DNA heterogeneity in protein-DNA co-condensates can generate forces up to piconewtons. This range of capillary forces is consistent with earlier findings on DNA condensation mediated by pioneer transcription factors such as Sox2 [10], and viral nucleocapsid proteins [91]. Meanwhile, molecular motors involved in chromatin organization, such as condensin and cohesin, can exert forces up to ∼1 piconewton on the DNA [92,93]. Interestingly, alterations in protein binding affinity at the single motif level, even by one *k*_*B*_*T* due to mutations or methylation patterns, can lead to two-fold changes in global forces. These forces have the potential to shape the three-dimensional structure of the genome. Moreover, sequence-dependent co-condensation of DNA and proteins can potentially form spatially heterogeneous chromosomal territories. Future studies can shed light on the feasibility of such a mechanism.

Overall, the protein-mediated condensation of DNA is increasingly recognized as a crucial mechanism for organizing the genome and responsible for other cellular processes [9,11,94]. Nevertheless, a comprehensive theoretical understanding remains in its early stages. In this light, our modeling framework can be harnessed to gain mechanistic insights into biophysical principles governing the sequence-dependent co-condensation of protein and DNA, and pave the way for developing a deeper understanding of genome organization.

## 5 Materials and methods

### 5.1 Limitations of the model

While our model offers insights into how sequence heterogeneity regulates the interfacial physics of DNA-protein co-condensation, it has certain limitations. For instance, our model does not take into account the changes in the mechanical properties of the DNA due to protein binding. Previous studies have shown that proteins from the high mobility group (HMG) family bend DNA upon binding, leading to the assembly of nucleoprotein complexes [95,96]. However, our model does not differentiate between the bending rigidity of DNA in its protein-bound and unbound states; we consider the persistence length of DNA to be 150 bp in both cases. It is important to note that although the magnitudes of forces observed in our simulations fall within the range reported in experiments, they may not precisely reflect those measured in specific *in vitro* studies. Achieving a more precise and quantitative agreement between model-predicted and experimentally observed forces may require detailed consideration of protein binding properties specific to the protein of interest. Nonetheless, our simplified model captures the complex influence of DNA sequence heterogeneity on the physics of protein-DNA co-condensation. Moreover, we carry out Brownian dynamics simulation to study the kinetic and equilibrium properties of the system. Although Brownian dynamics simulations are slower than Monte Carlo methods for sampling equilibrium properties, we choose the former because our focus is on both the equilibrium and the kinetics of DNA-protein co-condensation. Alternatively, we could have employed the kinetic version of the Monte Carlo algorithm, as employed by Tortora et al. [23]. For example, the authors showed that the idealized mouse chromosome 19 model in Ref. [23], exhibits a coarsening kinetic exponent of $R∼t^{1/2}$ (Figure 4E in [23]). This model of mouse chromosome 19 has a chromatin (polymer) sequence with a continuous high binding affinity region of HP1 (protein). Our heterogeneous DNA I model is similar to Tortora et al.’s model in terms of sequence heterogeneity and shows the same scaling exponent of $R∼t^{1/2}$ (Fig 8). Although our model operates at a different scale—where one monomer of the DNA represents 10 base pairs compared to 1 kilobase pair per monomer in Ref. [23]—we find good agreement in the coarsening exponents, capturing the qualitative features of the kinetics. Overall, our study demonstrates that sequence heterogeneity dictates capillary forces as well as impacts the coarsening kinetics of DNA-protein condensates.

Our model of protein-DNA co-condensation is designed to capture and explain experimental observations typically made using optical tweezers and coverslip-based assays, which commonly employ simple duplex DNA substrates. However, DNA in physiological contexts is organized into nucleosomes and higher-order chromatin structures. *In vitro* studies involving nucleosomes remain limited, with recent work by Nguyen et al. [10] providing important insights. They found that in nucleosomal configurations, where DNA is wrapped around histone octamers, capillary forces are significantly reduced. Nguyen et al. [10] experimentally demonstrated that the presence of histones reduces the capillary forces exerted by Sox2-DNA condensates by approximately half compared to naked DNA. These experimental findings highlight the need for theoretical models that explicitly incorporate nucleosomes. While our model offers important biophysical insights into how naked DNA sequence influences protein-DNA co-condensation, extending it to include nucleosomal and higher-order chromatin structures will be important for accurately capturing *in vivo* physiological conditions. Future studies should systematically explore these effects to deepen our understanding of chromatin organization.

### 5.2 Parameter selection

Recent experimental studies on protein–DNA co-condensation report that various DNA-binding proteins undergo phase separation over a concentration range of approximately 2.5 μM to 200 μM [7,80,97]. For example, FUS proteins alone phase-separate at around 2.5 μM concentration [97], whereas Lsr2 and nPhos–HP1α (the phosphorylated, functional form of HP1α) exhibit phase-separation thresholds of 5 μM and 192 μM, respectively [7,80]. Importantly, the addition of DNA substantially lowers these threshold concentrations, shifting them into the range of 0.1 μM to 50 μM; for instance, FUS–DNA co-condensation occurs at approximately 100 *nM*, while Lsr2–DNA and HP1–DNA co-condensates form at roughly 500 *nM* and 50 μM, respectively. To capture these experimental observations, we set the protein–protein interaction strength to 2 *k*_*B*_*T*, which yields protein-only phase separation near 190 μM. Moreover, protein–DNA interaction strengths in our study range from 0.1 *k*_*B*_*T* to 4 *k*_*B*_*T* across different models. In particular, a protein–DNA interaction strength of 2 *k*_*B*_*T* leads to co-condensation at approximately 40 μM (see Fig F in S1 Text), ensuring that our simulations operate within a physiologically relevant regime. Moreover, the parameter values we employ are consistent with previous studies [7–9,17,20,22,24,26,80,98]; for example, Takaki et al. [98] calibrated protein interaction strengths using single-molecule measurements from FoxA1–DNA condensation by Quail et al. [11] and found that an interaction strength of 2 *k*_*B*_*T* sufficiently captures the experimental behavior, aligning with the values adopted in our work. Table 1 summarizes the model parameters and their agreement with previous studies.

### 5.3 Simulation methodology

We employ Brownian dynamics to simulate the protein-DNA system using ESPResSo package [99]. The temperature of the system is set to 1.0 *k*_*B*_*T* using Langevin thermostat whereas the damping coefficient is set to 0.1 $\tau^{-1}$. We use a time step of Δt=0.01 τ to integrate the equations of motion. Please refer to Sec. S1 in S1 Text of the Supplementary Information (SI), including Table A-D, for details of the model.

We start with a semiflexible polymer in a box of dimensions(x, y, z) =(80 σ, 80 σ, 600 σ), where σ is the size of one monomer. Guided by optical tweezer experiments, we fix the ends of the polymer to maintain a constant end-to-end distance (Fig A in S1 Text, top panel). We equilibrate the polymer in the absence of proteins. Next, We save five uncorrelated DNA configurations which are used as initial configurations for simulations in the presence of proteins. For each of these independent realizations, we introduce proteins in the simulation box (Fig A in S1 Text, middle panel), and integrate the system till equilibrium is ensured (Fig A in S1 Text, bottom panel). The equilibration time varies for the DNA models considered in our study, end-to-end distance, and protein concentration. After equilibration, we run the system for $1.5\times 10^{7}$ steps and save 3000 independent configurations for all subsequent analyses. All images are created using Visual Molecular Dynamics (VMD 1.9.3) [100]. Note that the field of view of the snapshots is adjusted to highlight specific features, which may result in variations in image dimensions.

### 5.4 Condensate detection and volume analysis

We use the Density-Based Spatial Clustering of Applications with Noise (DBSCAN) to identify condensates in our simulations [101]. DBSCAN depends on two critical parameters: epsilon (ε) and Minimum Points. The parameter epsilon (ε) defines the radius around each particle, within which neighboring points are considered part of the cluster. The other parameter, Minimum Points, specifies the minimum number of points within this radius required to form a cluster. In our study, we set Minimum Points to 6, following the common practice of using twice the dimensionality of the dataset (here, 3D coordinates of particles) [101]. To make use of the DBSCAN algorithm for identifying the condensates, it is essential to determine the optimal value for epsilon (ε). To do this, we follow the standard approach which is to calculate the distance between each point and its k-th nearest neighbor (in our study, k is set to 5; k = Minimum Points –1 [102]). Sorting and plotting these distances result in a k-distance plot (see Fig B, panels A and B in S1 Text) revealing any density-based grouping present in the data. The point of maximum curvature, known as the “knee point”, serves as a good estimate for epsilon (ε) [102].

We start by plotting the k-distance plot using the distance of each data point from its 5th nearest neighbor (see Fig B, panel A in S1 Text, red curve). However, due to noise in the data, the epsilon value obtained from this raw plot is inaccurate (see Fig B, panel A in S1 Text, red point). We plot a zoomed-in view of the grey-shaded region shown in plot A to better illustrate the fluctuations present in the raw data (see Fig B, panel B in S1 Text, red curve). When this ε value is used as the input parameter in DBSCAN, the monomers outside the condensates are erroneously classified as part of the condensate (see Fig B (i) in S1 Text).

To address this issue, we apply the Savitzky-Golay filter [103], a digital filter commonly used for signal smoothing. This filter fits polynomials of varying degrees to consecutive data points within a moving window of a specified length. In our study, we use third-degree polynomials with window lengths of 99 (see Fig B, panels A and B in S1 Text (green curves)) and 199 (see Fig B, panels A and B in S1 Text (blue curves)). When the ε value is estimated by using the Savitzky-Golay filter with window lengths of 99 and 199, and then applied in the DBSCAN algorithm, we observe different outcomes. With a window length of 99 (see Fig B (ii) in S1 Text), the algorithm still fails to accurately detect the condensate. In contrast, ε value estimated with window length 199 correctly detects the particles present in the condensates (see Fig B (iii) in S1 Text). We repeat this procedure for all the configurations in each simulation to identify the condensates, which are then used for force, volume, and interfacial affinity analysis.

To compute the volume of the condensates, we use the maximum pairwise distance between the particles identified as part of the condensate along all three axes. We define the condensate radius along each axis as half of the maximum distance. Volume is then calculated as


$$V_{c}=\frac{4}{3}\pi \times r_{x}\times r_{y}\times r_{z}.$$ (1)

Here, $V_{c}$ is the volume of the condensate, *r*_*x*_, *r*_*y*_, and *r*_*z*_ are condensate radii along the three axes.

### 5.5 Force and potential energy analysis

To calculate the force due to condensate, we first find the force due to tethering the DNA at the specified $R_{e}^{\prime }$, and subtract this from the force calculated in the presence of interacting proteins. This step distinguishes the intramolecular forces acting on DNA beads from other DNA beads, from the intermolecular forces exerted by proteins present in the system.

(i) For force calculation in the absence of proteins, we compute bond stretching due to fixing the ends of the polymer. We calculate the change in bond lengths of all 499 bonds present in the polymer. As expected, the distribution of bond length for the tethered ends is identical to the rest of the bonds (Fig C, panel A in S1 Text). We calculate the force on polymer in the absence of proteins as


$$F_{DNA}=\langle k_{b}\left(\overset{―}{l_{t}}-\overset{―}{l_{0}}\right)\rangle .$$ (2)

Here, *k*_*b*_ is the harmonic bond strength, $\overset{―}{l_{t}}$ is the mean bond length of the DNA with tethered ends, and $\overset{―}{l_{0}}$ is the mean bond length of the DNA with free ends. The angular brackets in the above equation denotes the average over 3000 independent configurations. We calculate the force for different normalized end-to-end distances ($R_{e}^{\prime }$). Fig C, panel B in S1 Text shows a marginal change in *F*_*DNA*_ up to $R_{e}^{\prime }$ = 0.8, followed by a sharp rise at $R_{e}^{\prime }$ = 1, and is qualitatively consistent with the theoretical prediction of the worm-like chain (WLC) model [15].

(ii) For force calculation due to protein-DNA co-condensates, we first identify the condensates using DBSCAN. Next, we calculate the mean bond length of monomers outside the condensate ($\overset{―}{l_{t,p}}$) and calculate the force as,


$$F_{DNA,protein}=\langle k_{b}\left(\overset{―}{l_{t,p}}-\overset{―}{l_{t}}\right)\rangle .$$ (3)

We average the force over 3000 equilibrated configurations. The error bars in the force-extension curves are standard deviations over 5 different initial conditions. Finally, we find the force due to condensation as,


$$F=F_{DNA,protein}-F_{DNA}.$$ (4)

Fig C, panel C in S1 Text shows *F*_*DNA*,*protein*_ and *F* for a homogeneous DNA as a function of $R_{e}^{\prime }$ to illustrate the procedure.

(iii) To find the potential energy of each monomer along the DNA contour, we start with computing potential energy contributions from monomer-protein interactions and monomer-monomer interactions. We then add these contributions from different interactions to calculate the potential energy for each monomer of a configuration (Fig D in S1 Text). We observe a sharp decline in the potential energies of the monomers at the left interface and a sharp rise in the potential energy at the right interface of the condensate.

### 5.6 Interfacial affinity analysis

After identifying the condensate for each configuration using DBSCAN, we assign a score (*s*_*m*,*c*_) of 1 or 0 to each monomer *m* if it is inside or outside the condensate in *c*_*th*_ configuration. We find the probability of monomer *m* to be inside the condensate (*P*_*m*_) by,


$$P_{m}=\frac{\sum_{c=1}^{\Omega_{c}}s_{m,c}}{\Omega_{c}},$$ (5)

where, $\Omega_{c}$=3000, is the number of equilibrated configurations chosen for the analysis. We consider the monomers to be part of the interface if $0.1<P_{m}\leq 1$ (as shown in Fig E in S1 Text). Finally, we calculate the interfacial affinity (*I*_*a*_) by,


$$I_{a}=\frac{\sum_{m=1}^{N_{mI}}\varepsilon_{mp}P_{m}}{\sum_{m=1}^{N_{mI}}P_{m}},$$ (6)

where $\varepsilon_{mp}$ is the interaction strength between monomer and proteins, and *N*_*mI*_ number of monomers within the interface. In every independent realization, we compute the two interfacial affinities corresponding to each interface of the condensate and report their average.

## Supporting information

S1 Text Model details, parameters and supplementary figures. (PDF)
