# Supplementary material for: Decoding the role of DNA sequence on protein-DNA co-condensation
Source: PLoS Comput Biol. 2025 Dec 18;21(12):e1013829. doi: 10.1371/journal.pcbi.1013829 (PMC12725741; doi:10.1371/journal.pcbi.1013829)
Supplement: S1 Text — (PDF) [file pcbi.1013829.s001.pdf]

Decoding the role of DNA sequence on protein-DNA co-condensation

Rohit Kumar Singh[1,2 &], Pinaki Swain[1,2 &], Mahipal Ganji[3], Sandeep Choubey [1,2 *]

[1]The Institute of Mathematical Sciences, CIT Campus, Tharamani, Chennai, India
[2]Homi Bhabha National Institute, Training School Complex, Anushaktinagar, Mumbai, India
[3]Department of Biochemistry, Indian Institute of Science, Bangalore, India

1 Model details

We use a minimal model to study the protein-DNA co-condensation on end-tethered DNA. We model the DNA as a coarse-grained semiflexible polymer consisting of 500 monomers, and the proteins (P) as spherical particles with the same size as the monomers. Each monomer (M) in the model maps to 10 base pairs(bps) of DNA, which implies that each protein binds to 10 bps of DNA. Harmonic springs link the adjacent monomers, whereas harmonic angle potential between consecutive bonds models the semi-flexibility of DNA. The bond length potential is given by,

$$U(r) = \frac{1}{2}k_b(r - r_0)^2. \tag{1}$$

Here, k_b and r_0 are the bond length energy and equilibrium bond length, respectively. We set $k_b = 100\, k_BT/\sigma^2$ and $r_0 = \sigma$. The bond angle potential is given by,

$$U(\theta) = \frac{1}{2}k_\theta(\theta - \theta_0)^2. \tag{2}$$

Here, k_θ and θ_0 are the bond angle energy and equilibrium bond angle. We set $k_\theta = 15\, k_BT$ and $\theta_0 = 180°$. k_θ relates to the persistence length(l_p) of DNA as,

$$l_p = \frac{k_\theta}{k_BT}. \tag{3}$$

We consider four different DNA sequences in the study. In each of the sequences, DNA is modeled as a self-avoiding polymer with only repulsive interaction between the monomers. To model the repulsion between the monomers, we use Weeks-Chandler-Anderson (WCA) potential[1], which is a purely repulsive form of Lennard-Jones potential. To model the non-bonded interactions among monomer-protein and protein-protein, we use the standard Lennard-Jones potential with attractive tail.

$$U_{nb}(r_{ij}) = \begin{cases} 4\epsilon_{ij}\left[\left(\frac{\sigma}{r_{ij}}\right)^{12} - \left(\frac{\sigma}{r_{ij}}\right)^{6} + \frac{1}{4}\right], & r_{ij} \le r_{cut} \\ 0. & r_{ij} > r_{cut} \end{cases} \tag{4}$$

where r_{ij} is the distance among the particles i and j.

However, the details of the monomer-protein and protein-protein interactions vary for different sequences. For the homogeneous DNA (Fig 2A) which consists of only one type of monomers (A), we have three interacting pairs, namely, AA, AP, and PP. As stated above, we keep purely repulsive interaction between AA pairs to model the volume exclusion of monomers. To begin with, we keep the strength of monomer-protein attraction (ϵ_{AP}) and as well as protein-protein attraction (ϵ_{PP}) equals to 2 k_BT. Table A summarizes the non-bonded interaction parameters for homogeneous DNA-protein co-condensate system.

ij	ϵ_{ij} (k_BT)	r_{cut} (σ)
AA	1.00	$2^{\frac{1}{6}}$
AP	2.00	2.5
PP	2.00	2.5

Table A: Non-bonded interaction parameters for all the particle pairs in the homogeneous DNA-protein condensate system: A represents the monomers, and P represents the proteins.

For the heterogeneous DNA I (Fig 2B), we consider the DNA to be made up of two types of monomers, A and B. While A monomers attract the protein P with 1.75 k_BT strength, B monomers attract the proteins P with 2.25 k_BT strength. The sequence (Fig 2B) has a -AAAAA-BBBBBBBBBB-AAAAA- like architecture with the central 250 B monomers having a stronger affinity for the proteins P. The central B region is flanked by two stretches of A monomers each of length 125, having a weaker affinity for the proteins P. The interaction parameters for heterogeneous DNA model I are summarized in Table B.

ij	ϵ_{ij} (k_BT)	r_{cut} (σ)
AA	1.0	$2^{\frac{1}{6}}$
AB	1.0	$2^{\frac{1}{6}}$
BB	1.0	$2^{\frac{1}{6}}$
AP	1.75	2.5
BP	2.25	2.5
PP	2.0	2.5

Table B: Non-bonded interaction parameters for all particle pairs. A, and B are monomers and P represents the proteins. Monomer A has a weaker affinity for the proteins (1.75 k_BT) and monomer B has a stronger affinity for the proteins (2.25 k_BT).

For the Heterogeneous DNA II (Fig 4A, Fig J, panels A and B), the sequence has a blocky architecture arranged as -AAAAA-BBBBB-AAAAA-BBBBB-AAAAA-. Each block consists of 100 monomers. The two blocks of high-affinity regions consisting of B monomers are separated by a low-affinity region made up of 100 A monomers. To examine how the DNA-protein and protein-protein interactions govern the coexistence of multiple condensates on the DNA, we consider three different interaction regimes for Heterogeneous DNA II. Across all regimes, A monomers interact with the protein P with 0.1 k_BT strength, for B monomers we set the DNA-protein interaction strength to 2.25 k_BT for regime 1 (Fig J, panel A) and regime 2 (Fig 4A), and 1.50 k_BT for regime 3 (Fig J, panel B). In contrast to the other models where protein-protein interaction strength is set to 2.0 k_BT, here, we set it to 1.5, 2.0, and 2.25 k_BT in regimes 1, 2, and 3 respectively. The interaction

parameters for heterogeneous DNA II model are summarized in Table C.

ij	ϵ_{ij} (k_BT)	r_{cut} (σ)
AA	1.0	$2^{\frac{1}{6}}$
AB	1.0	$2^{\frac{1}{6}}$
BB	1.0	$2^{\frac{1}{6}}$
AP	0.1	2.5
BP	1.5–2.25	2.5
PP	1.5–2.25	2.5

Table C: Non-bonded interaction parameters for all particle pairs. A and B are monomers and P represents proteins. Monomer A has a weaker affinity for the proteins (0.1 k_BT) and monomer B has a stronger affinity for the proteins (2.25 k_BT in regimes 1 and 2, and 1.5 k_BT in regime 3).

Our fourth DNA sequence is the last 5 Kbp stretch of λ-phage DNA (partial λ-DNA) sequence. We assume that the protein P binds to the AT-rich region of the sequence. Since one monomer in our model corresponds to 10 bps, each monomer may have a different affinity for the protein P, based on the AT content of the underlying 10 bp sequence. If all the 10 bps are either Adenine or Thyamine nucleotide, then it has 100% AT content, whereas if none of the sequences is A or T, then it has 0% AT content. So, the AT content of a 10 bps sequence can only have 11 possible values. For this purpose, we consider our system to be made up of 11 types of monomers (A, B, C, D, E, F, G, H, I, J, K), each corresponding to a different AT content of the 10 bps sequence. Using this formalism, we find the AT content of the partial λ-DNA as a function of the sequence length (Fig 5A). Next, we assign different monomer-protein affinities to these 11 monomers on a scale from 0.1 to 4 k_BT. We summarize the monomer-protein interaction parameters in Table D. We keep ϵ_{PP}= 2 k_BT as in the previous sequences.

AT content(%)	monomer (M)	ϵ_{MP} (k_BT)
0	A	0.1
10	B	0.4
20	C	0.8
30	D	1.2
40	E	1.6
50	F	2.0
60	G	2.4
70	H	2.8
80	I	3.2
90	J	3.6
100	K	4.0

Table D: AT content of 10 bp of eleven kinds of monomers in the partial λ-DNA model and their interaction affinities with protein P.

2 Supplementary figures

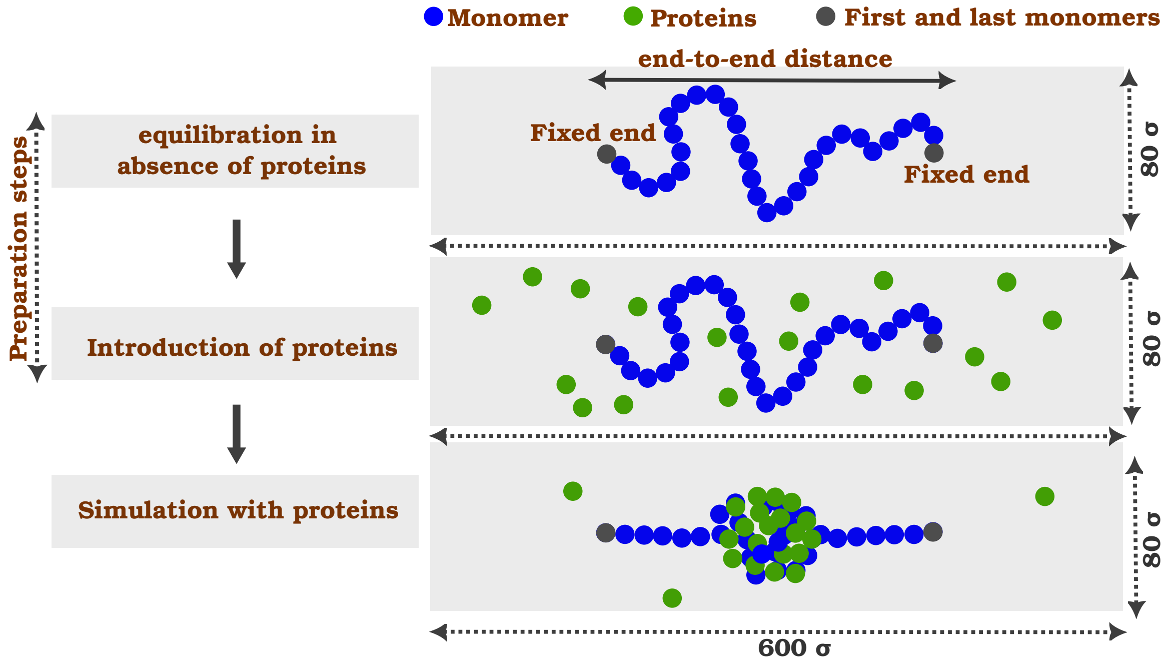

Fig A: A schematic representation of the simulation procedure: Polymer is placed in a box with a fixed end-to-end distance (top), proteins are introduced in the system after equilibrating the polymer (middle), and equilibration of DNA and proteins leads to formation of a cocondensate (bottom).

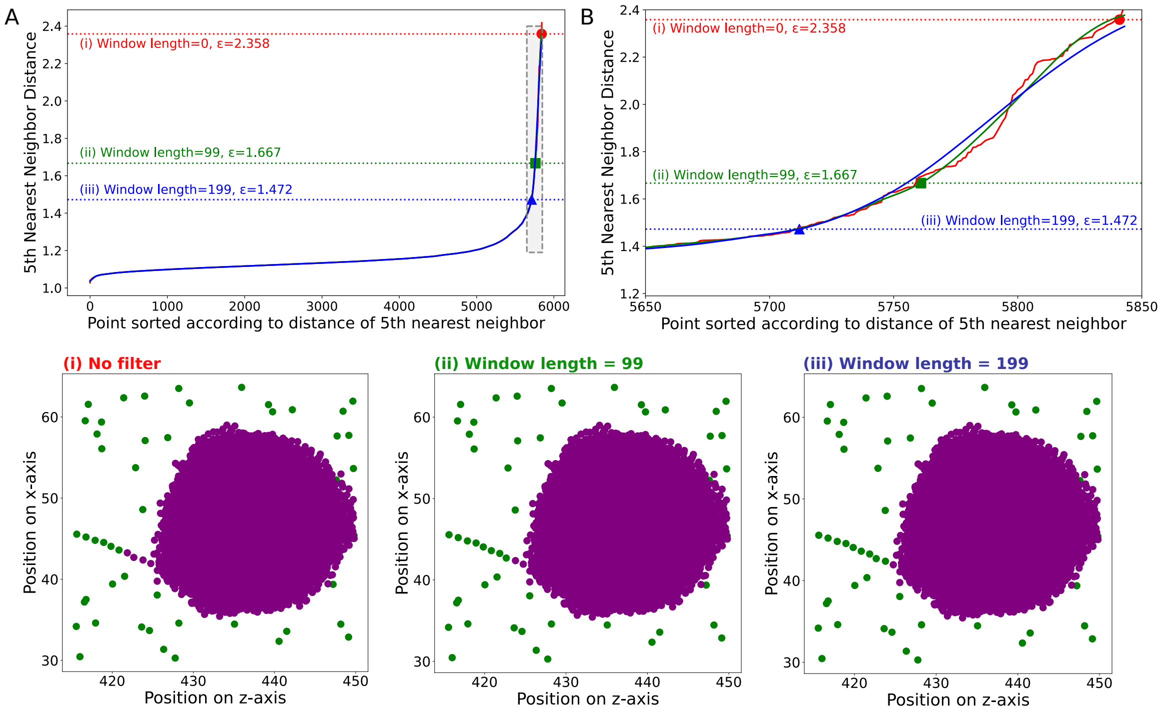

Fig B: Application of the DBSCAN algorithm in condensate detection: **A** The k-distance plot is shown for the raw data (red) and the smoothened curves with window size = 99 (green) and 199 (blue) for a configuration of homogeneous DNA. **B** A zoomed-in view of the shaded region in plot A. (i) The scatter plot shows the identified condensate region in the x-z plane for ε calculated from the raw data. The green points indicate regions in the dilute phase, while the purple points are part of the condensate. (ii) and (iii). The scatter plots of the same configuration using ε values calculated from the smoothed data for window lengths of 99 and 199, respectively.

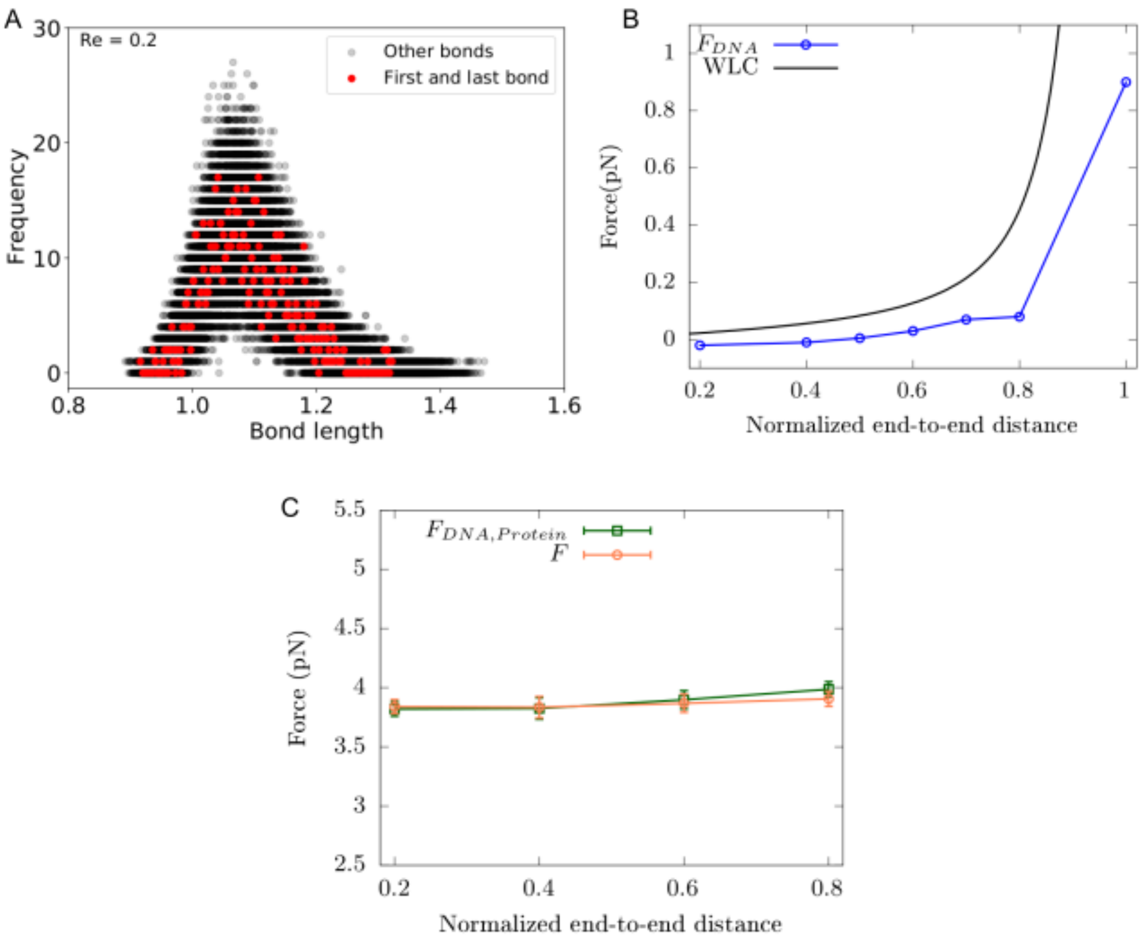

Fig C: **A** The bond length distribution for a tethered polymer in the absence of proteins is shown at R_e=0.2. Red points represent the distribution of bond length of the first and last bond. Points in black represent the bond length distribution of the remaining bonds. We use the average of black points to find $\overline{l_t}$. **B** Comparison of force-extension behavior of DNA in the absence of protein with the prediction of worm-like chain (WLC) model, $\frac{Fl_p}{k_BT} = \frac{1}{4(1-R_e')^2} - \frac{1}{4} + R_e'$. **C** Force due to DNA and proteins ($F_{DNA,protein}$) and only proteins (F) as a function of R_e' for the homogeneous DNA.

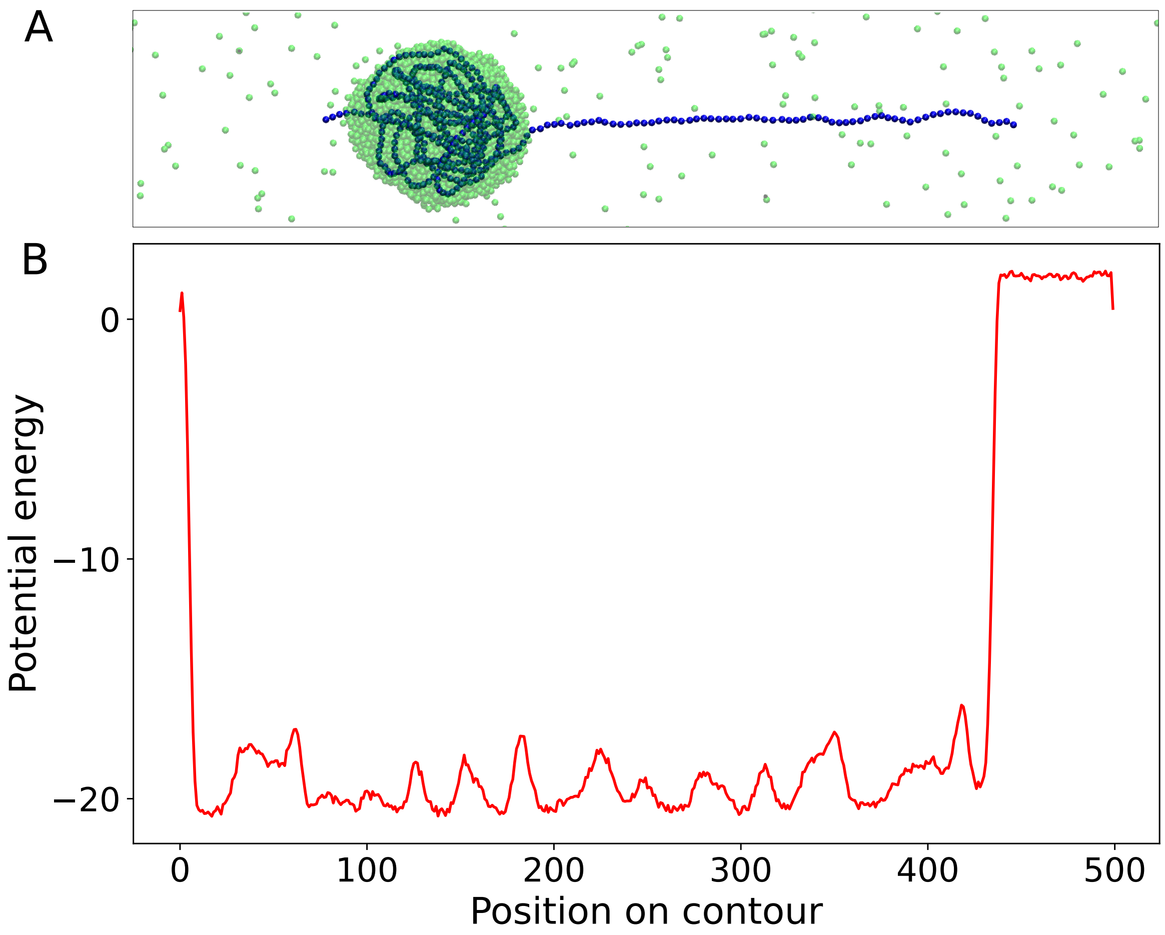

Fig D: **A** Representative equilibrium configuration of a homogeneous DNA (blue) in presence of proteins (green) at $R'_e = 0.2$ and $\rho_P = 84.50\ \mu$M. **B** Potential energy per monomer as a function of monomer position on the contour.

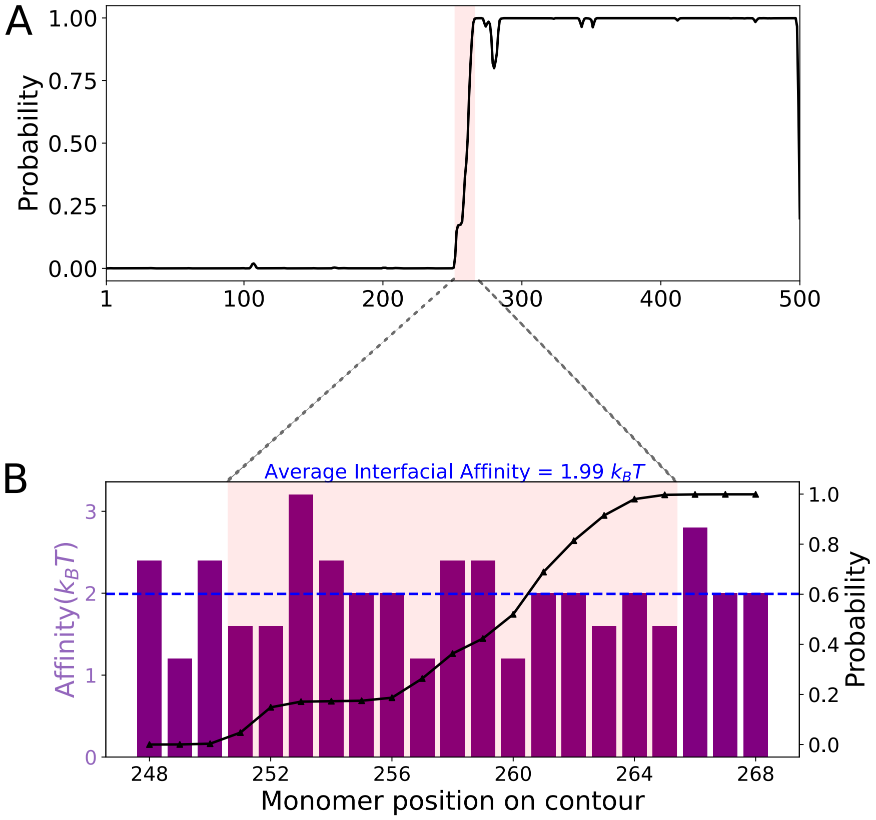

Fig E: **A** Probability of monomers to be in the condensate is plotted as a function of their position on the DNA contour for Partial λ-DNA at $\rho_p = 59.15\ \mu$M and normalized $R'_e = 0.6$. The red-shaded region represents the interface where the probability transitions from 0.1 to 1. **B** A zoomed-in view of the interface with the purple bars indicating the protein-binding affinity of the interfacial monomers.

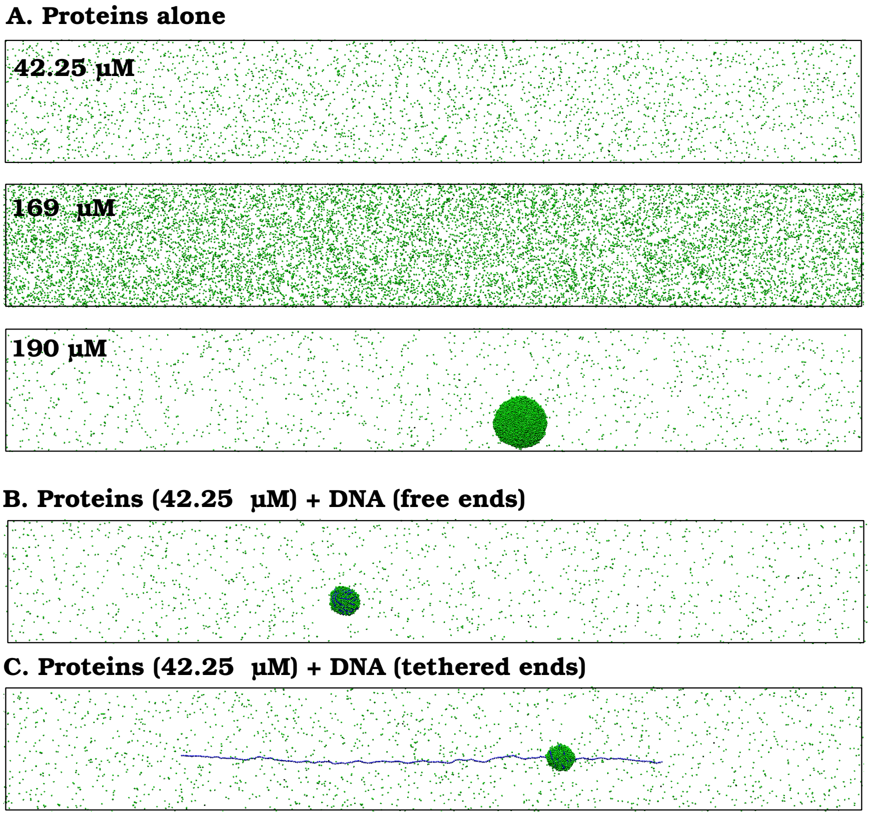

Fig F: **A** Equilibrium snapshots are shown at different protein concentrations in the absence of polymer. **B** Condensation of proteins and homogeneous DNA with free ends at equilibrium. **C** Condensation of proteins and homogeneous DNA with tethered ends ($R_e' = 0.6$).

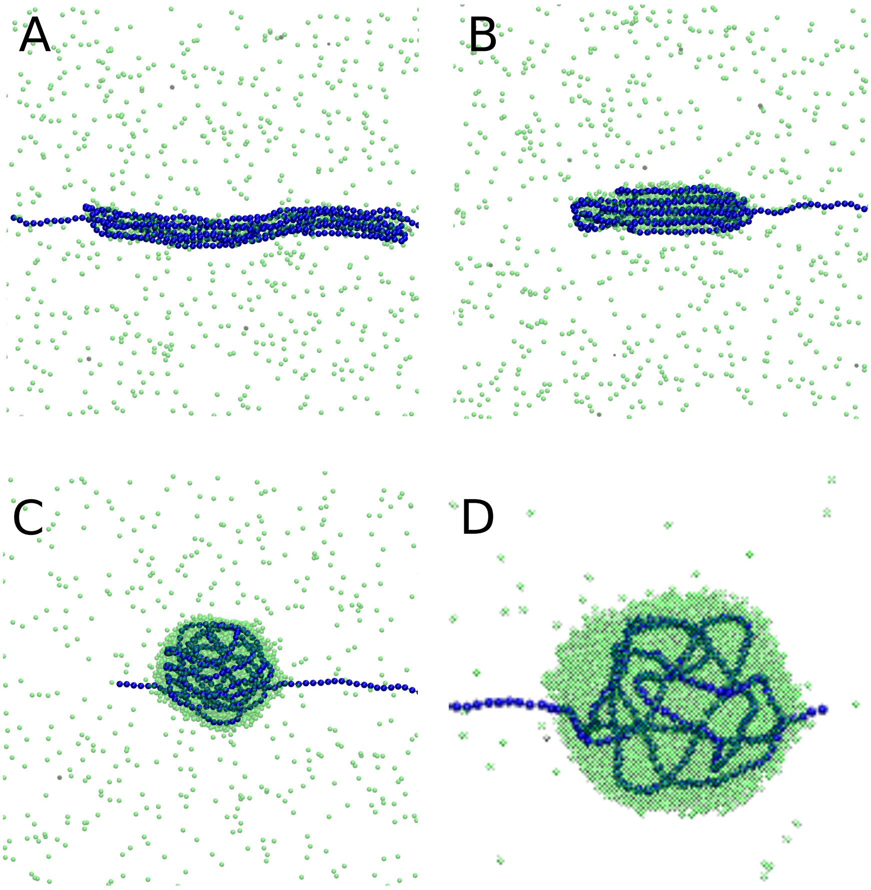

Fig G: Snapshots A, B, C, and D represent condensates at protein-protein interaction strength 1, 1.5, 1.8, and 2.0 k_BT, respectively at R'_e= 0.4 and ρ_p= 84.5 μM. DNA-protein interaction is 2.0 k_BT for all cases. Homogeneous DNA monomers are shown in blue and proteins are shown in green.

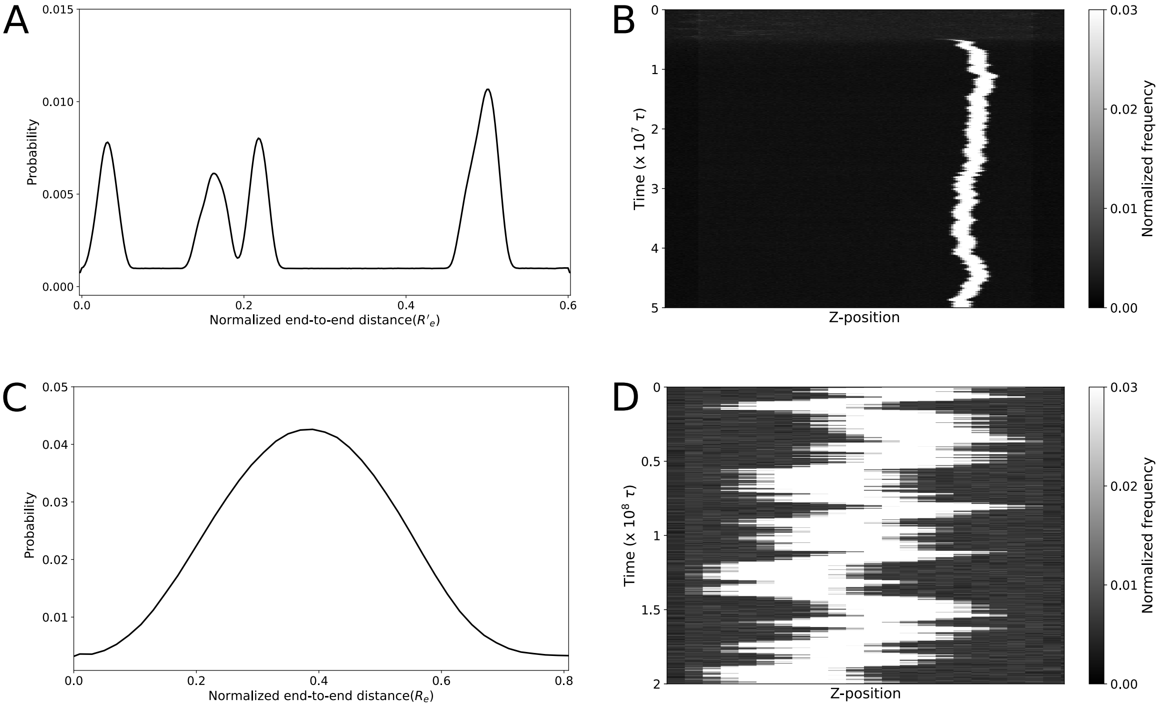

Fig H: **A** Histogram for distance between center of mass of the condensate and left tethered end is plotted for five independent replicates for homogeneous DNA (N_m =500 , l_p=15 σ) at $R'_e = 0.6$ and $\rho_p = 42.25\ \mu$M. **B** A representative kymograph showing the droplet position for one replicate in **A**. **C** Histogram for distance between center of mass of the condensate and left tethered end is plotted for 10 times longer simulations for a shorter, flexible homogeneous DNA (N_m =50, l_p=0) at R'_e = 0.8 and $\rho_p = \rho_p = 84.5\ \mu$M. **D** Kymograph corresponding to **C** shows that the droplet explores the entire length of the polymer.

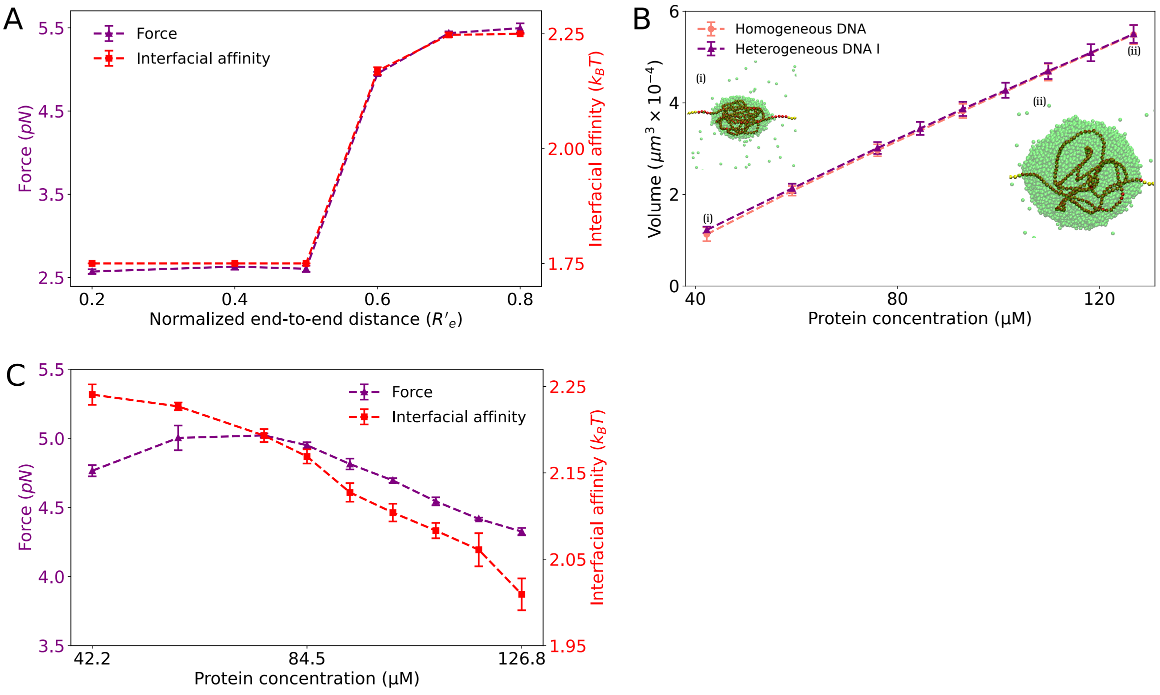

Fig I: **A** Force (purple) and interfacial affinity (red) as a function of R'_e is plotted across five independent realizations for heterogeneous DNA I at ρ_p=84.50 μM. **B** Volume of the condensate as a function of ρ_p for homogeneous DNA (salmon)and heterogeneous DNA I (purple). **(i)** and **(ii)** Condensate snapshots are shown for heterogeneous DNA I at ρ_p= 42.25 μM and ρ_p= 126.75 μM respectively. **C** Force (purple) and interfacial affinity (red) as a function of ρ_p at R'_e= 0.6 across five independent realizations for heterogeneous DNA I.

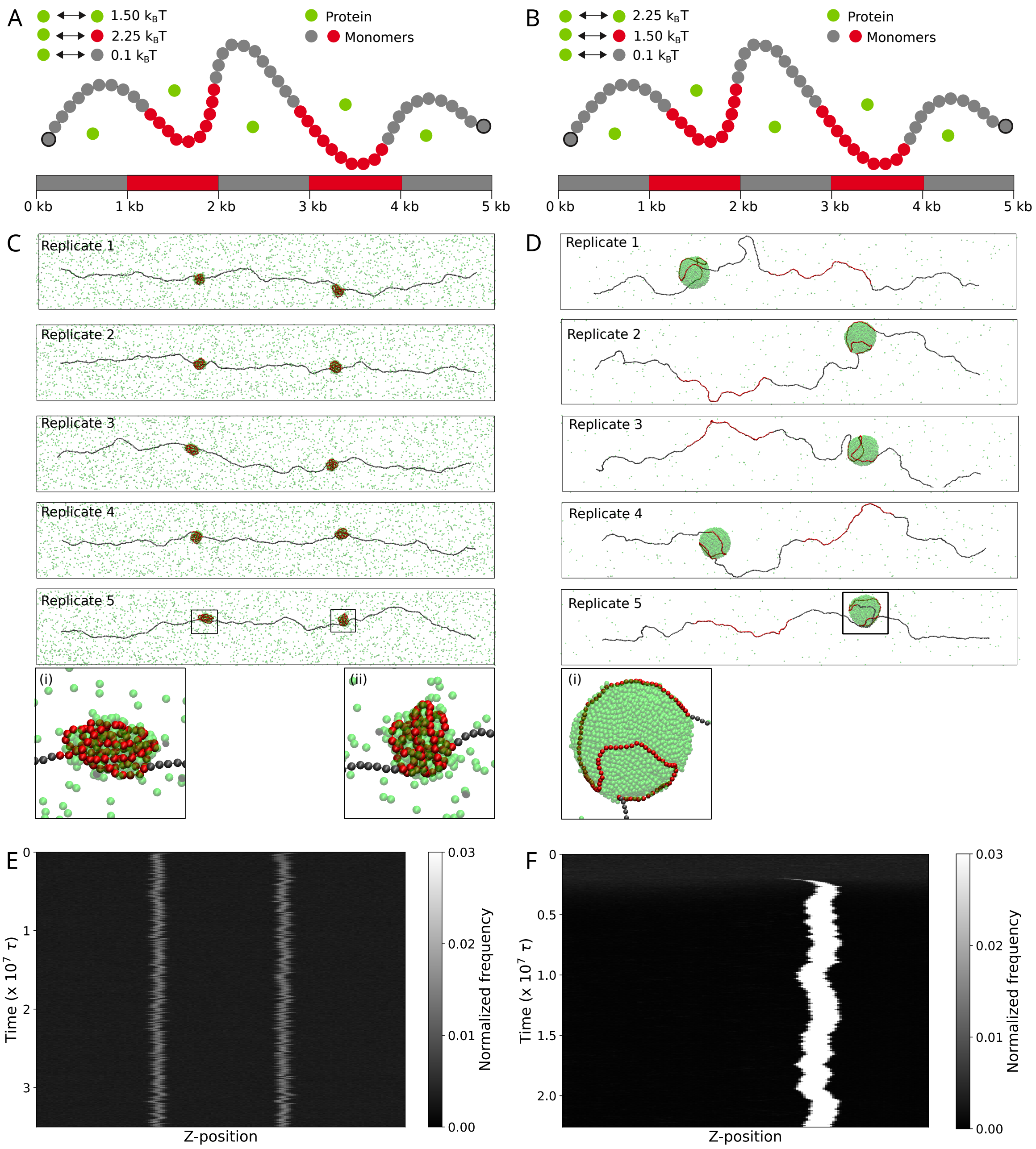

Fig J: **A** Schematic of heterogeneous DNA II with DNA-protein affinity higher than protein-protein affinity. Two blocks of high-affinity monomers (2.25 k_BT, 100 monomers in red) are separated by a block of low-affinity monomers (0.1 k_BT, 100 monomers in grey). Proteins (green) interact with each other with an interaction strength of 1.5 k_BT. **B** Schematic of heterogeneous DNA II with DNA-protein affinity lower than protein-protein affinity. Two blocks of high-affinity monomers (1.50 k_BT, 100 monomers in red) are separated by a block of low-affinity monomers (0.1 k_BT, 100 monomers in grey). Proteins (green) interact with each other with an interaction strength of 2.25 k_BT. **C** Snapshots of the system from (A) at $R_e' = 0.6$ and ρ_p=84.50 μM, showing two condensates co-existing at equilibrium for all five independent realizations. High-affinity monomers, low-affinity monomers, and proteins are shown in red, grey, and green respectively. (i) and (ii) Zoomed-in snapshots of the condensates form Replicate 5. **D** Snapshots of the system from (B) at $R_e' = 0.6$ and ρ_p=84.50 μM, with a single condensate at equilibrium for all five independent realizations. High-affinity monomers, low-affinity monomers, and proteins are shown in red, grey, and green respectively. (i) Zoomed-in snapshot of the condensate form Replicate 5. **E** A representative kymograph showing the droplet positions over time for replicate 5 in system (A). **F** A representative kymograph showing the droplet position over time for replicate 5 in system (B).

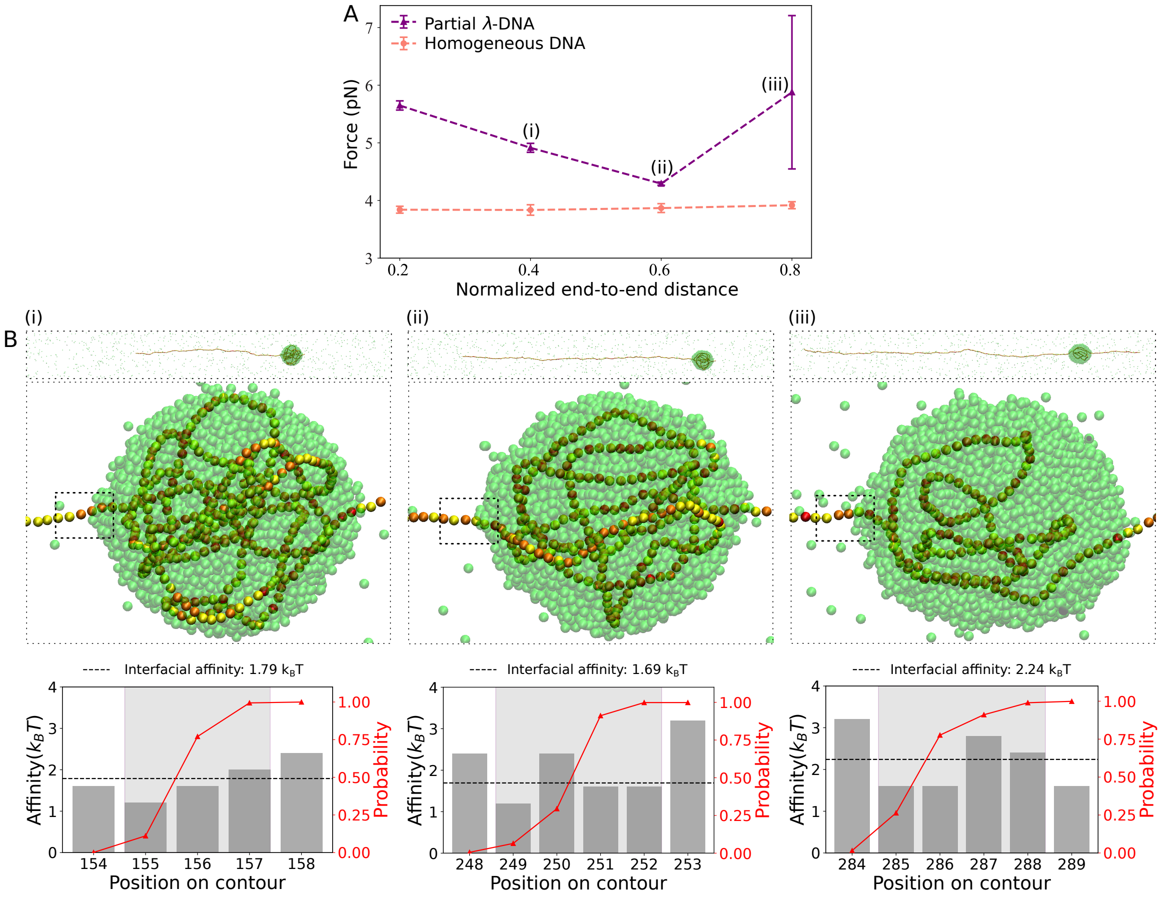

Fig K: **A** Capillary force as a function of R_e at fixed ρ_p (ρ_p=84.5 μM). **B** (top panel) Snapshots of condensates at concentrations marked as (i), (ii), and (iii) in **A**. Monomers are shown in yellow to red in ascending order protein binding affinity and the proteins are shown in green. **B**(bottom panel) Bar plots represent monomer-protein binding affinities. The grey shaded region shows the interface. The red curve shows the probability of monomer being inside the condensate.
